# Loss of the lysosomal lipid flippase ATP10B leads to progressive dopaminergic neurodegeneration and parkinsonian motor deficits

**DOI:** 10.1007/s00401-025-02908-0

**Published:** 2025-07-17

**Authors:** María Sanchiz-Calvo, Elena Coccia, Christopher Cawthorne, Gustavo Morrone Parfitt, Teresa Torre-Muruzabal, George Tsafaras, Koen Van Laere, Diego Cabezudo, Ana Cascalho, Chris Van den Haute, Peter Vangheluwe, Joel Blanchard, Eduard Bentea, Veerle Baekelandt

**Affiliations:** 1 Laboratory for Neurobiology and Gene Therapy, Department of Neurosciences, Leuven Brain Institute, KU Leuven, Leuven, Belgium; 2 Aligning Science Across Parkinson’s (ASAP) Collaborative Research Network, Chevy Chase, MD 20815 USA; 3 Nash Family Department of Neuroscience at Mount Sinai, New York, NY USA; 4 Friedman Brain Institute at Mount Sinai, New York, NY USA; 5 Nuclear Medicine and Molecular Imaging, Department of Imaging and Pathology, KU Leuven, Leuven, Belgium; 6 Laboratory of Cellular Transport Systems, Department of Cellular and Molecular Medicine, KU Leuven, Leuven, Belgium; 7 Leuven Viral Vector Core, KU Leuven, Leuven, Belgium

**Keywords:** Parkinson’s disease, In vivo, Nigrostriatal pathway, Dopaminergic neurons, ATP10B, PET, Behavior, Lysosomes

## Abstract

ATP10B, a transmembrane lipid flippase located in late endosomes and lysosomes, facilitates the export of glucosylceramide and phosphatidylcholine by coupling this process to ATP hydrolysis. Recently, loss-of-function mutations in the *ATP10B* gene have been identified in Parkinson’s disease patients, pointing to *ATP10B* as a candidate genetic risk factor. Previous studies have shown compromised lysosomal functionality upon *ATP10B* knockdown in human cell lines and primary cortical neurons. To investigate the role of ATP10B in Parkinson’s disease neuropathology, specifically in the nigrostriatal dopaminergic system, we induced ATP10B knockdown specifically in substantia nigra pars compacta neurons of rats using viral vector technology. Additionally, midbrain neuronal cultures derived from ATP10B knock-out human induced pluripotent stem cells clones were used to study the impact of ATP10B loss in dopaminergic neurons in a more translational model. *Atp10b* knockdown in rat brain induced parkinsonian motor deficits, and longitudinal striatal dopamine transporter ^18^F-FE-PE2I PET imaging revealed a progressive decrease in binding potential. Immunohistochemical analysis conducted one year post-injection confirmed the loss of dopaminergic terminals in the striatum, alongside a loss of dopaminergic neurons in the substantia nigra pars compacta. The expression of LAMP1, LAMP2a, cathepsin B and glucocerebrosidase was studied in dopaminergic neurons. A decrease in lysosomal numbers and an increase in lysosomal volume were observed more consistently in one of the knockdown constructs. The vulnerability of dopaminergic neurons to ATP10B loss-of-function was also observed in midbrain neuronal cultures derived from ATP10B knock-out human induced pluripotent stem cells clones, which showed a significant reduction in TH-positive neurons. Taken together, our findings demonstrate that ATP10B depletion detrimentally impacts the viability of dopaminergic neurons both in vivo and in vitro. Moreover, a broader impact on the functionality of the nigrostriatal pathway was evidenced as rats with *Atp10b* knockdown exhibited motor impairments similar to those observed in Parkinson’s disease patients.

**Supplementary Information:**

The online version contains supplementary material available at 10.1007/s00401-025-02908-0.

## Introduction

Parkinson’s disease (PD) is the second most prevalent neurodegenerative disorder, with a notable increase in both incidence and prevalence observed over the past two decades. The clinical spectrum of PD is characterized by motor symptoms such as tremor, bradykinesia, rigidity and postural instability, although non-motor aspects also contribute to the disease, such as constipation, cognitive decline, depression, and pain, among others [3]. The primary factor behind the motor impairment observed in patients with PD is the gradual neurodegeneration of dopaminergic neurons in the substantia nigra pars compacta (SNpc) [12]. These neurons extend axons to the dorsal striatum (dSTR) releasing dopamine and establishing the nigrostriatal dopaminergic pathway. This pathway contributes to the basal ganglia circuits involved in action selection, modulation and learning [1, 23]. Previous research has consistently shown impaired dopamine release from nigrostriatal neurons in numerous models of PD, often preceding or occurring in the absence of neurodegeneration [6]. Pathologically, PD is further characterized by the accumulation of aggregated α-synuclein in Lewy bodies and Lewy neurites. These structures, found in the affected brain regions, present a distinctive crowded environment comprising various membrane components, including vesicular structures, dysmorphic organelles like mitochondria, and high lipid content [28]. Therefore, the pathophysiology of PD presents a complex interplay between α-synuclein aggregation, dysfunctional mitochondria and lysosomes, disturbed lipid homeostasis and impaired vesicle transport [15, 19].

PD presents a multifactorial etiology involving genetics, environmental factors and the aging process. In recent years, the PD research community has dedicated substantial efforts to identify novel genetic factors associated with the pathogenesis of this complex disease. Notably, a significant proportion of these newly discovered genes have emerged as key players in the endolysosomal pathway, shedding light on the central role of endolysosomes in the development and progression of PD [19]. In a cohort of Belgian patients with PD and dementia with Lewy bodies (DLB) compound heterozygous missense variants were identified in a lysosomal gene from the P4-ATPase family known as *ATP10B*, which was proposed as a novel candidate genetic risk factor in PD. Identified pathogenic variants cause loss of the ATPase activity and are situated in conserved and functionally important domains [16]. Although mutations in *ATP10B* have been identified in PD patients from other cohorts, these studies did not confirm that *ATP10B* rare variants are causally linked to PD or increase the susceptibility to present the disease [8, 22, 33, 42]. On the other hand, the incomplete penetrance of *ATP10B* mutations, together with their low frequency and the importance of the *cis/trans* phasing of the compound heterozygous variants, underscore the need to study large cohorts and validate the *cis/trans* configuration to obtain significant results [30–32].

ATP10B is a transmembrane lipid flippase located in late endosomes and lysosomes, coupling lipid export of glucosylceramide (GluCer) and phosphatidylcholine (PC) to ATP hydrolysis [16, 40]. GluCer is also the main substrate of the lysosomal enzyme glucocerebrosidase (GCase) encoded by *GBA*. Interestingly, mutations in GBA are one of the main genetic risk factors in PD [29]. Lipids are major structural and functional components of cellular membranes. The lipid composition varies not only between membranes of different organelles but also between the cytosolic and exoplasmic leaflets of a single membrane, creating an asymmetric distribution that is a vital characteristic. ATP-dependent flippases play a pivotal role in maintaining membrane asymmetry by facilitating the transbilayer movement of lipids, a process that is energetically unfavorable [5, 17]. Pathogenic *ATP10B* variants exhibit a strongly impaired ability to translocate PC and GluCer [16, 40]. In addition, ATP10B regulates the uptake of PC and affects hexosylceramide levels in human cancer cell models [40]. Lysosomal functionality is compromised after ATP10B knockdown (KD) in WM-115 melanoma cells, as well as in primary cortical neurons with ATP10B KD, where the loss of ATP10B also increases susceptibility to cell death. Interestingly, these phenotypes in human cell lines and mouse cortical neurons are exacerbated by exposure to environmental PD risk factors such as rotenone and MnCl_2_ [16]_._

While in vitro models have offered insights into the cellular role of ATP10B, the consequences of ATP10B loss-of-function on PD neuropathology, particularly related to the function of dopaminergic neurons in vivo, remain unclear. To address these questions, we have developed a rat model with targeted ATP10B KD in the SNpc neurons and investigated its impact on the nigrostriatal dopaminergic pathway.

## Materials and methods

Key resources are listed in Supplementary Table 1.

### Study design

**Fig. 1:**
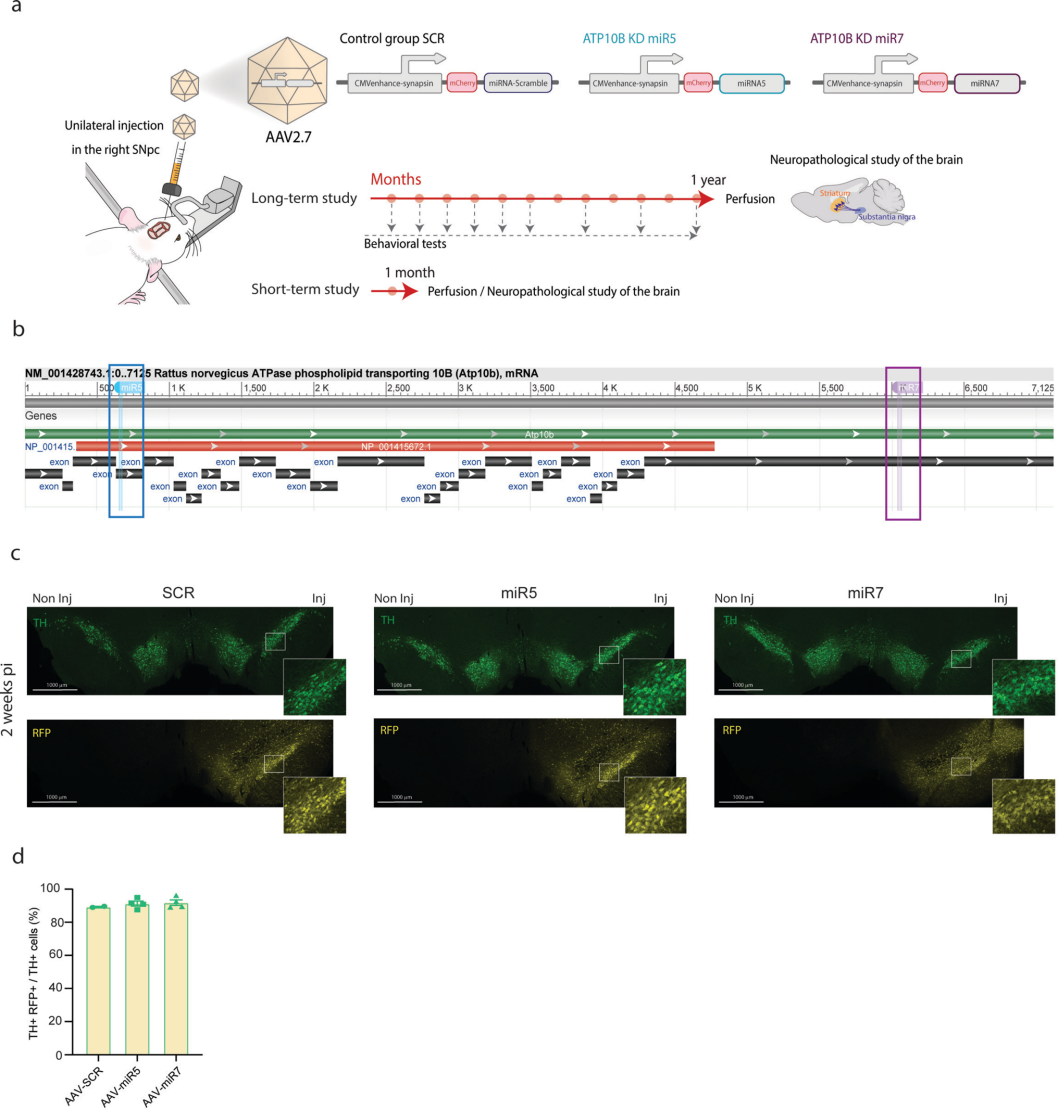
Targeted knockdown of ATP10B in adult rat SNpc using AAV vectors. miRNA-based short-hairpins (miR5 and miR7) targeting distinct regions of *Atp10b* mRNA, and a scrambled non-targeting sequence (SCR) packaged in AAV2/7 vector under a neuronal promoter (CMVenhanced-synapsin) were stereotactically injected in the right SNpc of rats. **a** Overview of the protocol followed in this study and vector constructs. **b** Schematic representation of the Atp10b mRNA sequence, with the full mRNA sequence shown in green, coding sequence (CDS) in red, and exons indicated in black. The binding sites for miR5 and miR7 are highlighted in blue and purple, respectively. **c**,** d** The percentage of double TH + RFP + cells within the total TH + population in the SNpc was determined for the three vectors 2 weeks post-injection. **c** Representative image of the SNpc of a rat injected with SCR, miR5 and miR7 vector 2 weeks post-injection, fluorescently stained with TH and RFP antibodies, shows efficient transduction of dopaminergic neurons. **d** Each data point represents an individual animal, and the number of TH + and TH + RFP + cells from 7 sections was quantified using stereological methods

Two miRNA-based short-hairpins (miR5 and miR7) targeting distinct regions of rat *Atp10b* mRNA (regions targeted on *Atp10b* mRNA miR5: 5’ TCCTGGTGATTCTGAACTGGAT 3’, miR7: 5’ TCCTAAGACAGTGCCTATATAT 3’) were packaged in an adeno-associated vector (AAV2/7) under a neuronal promoter (CMVenhanced-Synapsin) and unilaterally injected in the right SNpc of adult female Wistar rats. We have previously reported that these two miRNA-based short-hairpins efficiently KD ATP10B in mouse cortical neurons cultures [16]. A vector with a scrambled sequence (SCR: 5’ AATACGACGGTAAGTGAGTACG 3’) that has no equivalent target in the *Rattus norvegicus* genome was used as control. The AAV2/7 viral vectors were produced by the Leuven Viral Vector Core [36]. The long-term study consisted of 15–16 rats per treatment group. Following stereotaxic surgery, behavioral evaluation and in vivo striatal dopamine transporter (DAT) binding using ^18^F-FE-PE2I and positron emission tomography imaging (PET) were performed. Rats were euthanized 1 year post-injection to investigate neuropathological changes in the brain or changes in protein expression. A separate cohort consisted of 6 rats per treatment group and were sacrificed after 1 month without behavioral evaluation to examine possible short-term alterations by immunohistochemistry. Additionally, a smaller group of rats (2–4 per treatment group) were euthanized after 2 weeks to assess the efficiency of vector transduction in dopaminergic neurons. An overview of the study design is shown in Fig. 1.

### Animals

Adult (8–9 weeks old) female Wistar rats weighing 200–250 g (Janvier, France) were used for this study, and housed (2–3 animals per cage) in individually ventilated cages with free access to food and water, under a normal 12 h-light/12 h-dark cycle. Animal experiments were carried out in accordance with the European Communities Council Directive of November 24, 1986 (86/609/EEC) and approved by the Bioethical Committee of the KU Leuven (Belgium) (ECD project 161/2022). As a limitation, findings reported in this manuscript relate to female rats, as male rats were not included in this study. Similar experiments in male rats would be important to generalize our findings to both sexes.

### Stereotaxic surgery

All surgical procedures were performed using aseptic techniques. Rats were anaesthetized with ketamine (60 mg/kg, intraperitoneal (i.p.), Nimatek, Dechra, Belgium) and medetomidine (0.4 mg/kg, i.p., Domitor, Orion Pharma, Finland), and placed in a stereotactic frame (Stoelting, Wood Dale, IL). Rats were injected unilaterally with 3 µL of AAV2/7-CMVenhsynapsin-mCherry-miRSCR, AAV2/7-CMVenhsynapsin-mCherry-miR5, or AAV2/7-CMVenhsynapsin-mCherry-miR7 in the right SNpc (AP: – 5.3 mm; L: – 2.0 mm; DV: – 7.2 mm calculated from dura, using bregma as reference), using a 30-gauge needle and a 10 μl Hamilton syringe (Hamilton, Bonaduz, GR, Switzerland). Each vector was injected at a normalized titer of 7.5 × 10^11^ GC/mL (2.25 × 10^9^ GCs injected per animal), at a flow rate of 0.25 μL/min. Following injection, the syringe was left in place for an additional 5 min, and then slowly retracted.

### Behavioral tests

Different behavioral tests were performed to assess motor asymmetry and motor function. For each test, rats were acclimatized to the testing room at least 1 h prior to assessment. Automated systems were used for the rotarod and open-field tests. Quantification of the videos for the other tests was done by a blinded researcher.

#### Rotarod test

Motor coordination and balance was assessed using an accelerated rotarod system (IITC Life Science Rotarod Model I-755, Campden Instruments). Before surgery, rats were trained for 5 min at a constant speed of 5 rpm. During this initial training phase, rats were placed back on the rod after falling. In the second phase of training, rats underwent 3 trials of 1 min at a fixed speed of 5 rpm, 10 rpm, and 15 rpm, with 5 min of rest in-between trials. For testing rotarod performance at baseline, and post-surgery, rats were positioned on rotating rods with a progressively increasing rotation speed, ranging from 4 to 40 rpm over a 5 min period. Three trials were conducted at every timepoint with 5 min resting interval in between. The rotarod protocol was performed on three consecutive days, with the average of the three days taken for statistical analyses.

#### Open field test

The open field set-up consisted of a square box (1 m × 1 m) surrounded by opaque walls that prevent observation of visual cues outside the arena. Rats were placed in the center and spontaneous behavior was recorded using an overhead camera for 5 min. Total distance traveled, velocity, and % immobility time were obtained using AnimalTracker [9], and ipsi-/contralateral 180° turning and rearing frequency were calculated manually in a blinded manner on the acquired videos.

#### Cylinder test

The cylinder test was employed to quantify asymmetry in forelimb use. Contacts made by each forepaw with the wall of a 20-cm-wide clear glass cylinder were scored from the videotapes by an observer. A minimum of 20 contacts were recorded and quantified for each animal. The number of contralateral forelimb contacts was expressed as a percentage of total forelimb contacts. Rats not performing enough contacts were excluded from the analysis.

#### Elevated body swing test

In the elevated body swing test (EBST), the rat tail was taken approximately 3 cm from its base, and the animal was elevated to about 5 cm above the cage floor and held along the vertical axis for 5 s. Ipsilateral or contralateral swing were counted manually in a blinded manner, whenever the animal moved its head out of the vertical axis to either side by at least 30° within the 5 s observation interval. The EBST comprised five trials of maximum 5 s, each followed by a brief rest. The mean for all five trials was used for statistical analysis.

#### Catalepsy test

The catalepsy bar test is used to measure the failure to correct an imposed posture resulting from muscular rigidity. For this test, the forepaws of the rats were placed on an elevated bar with the hind paws remaining on the floor. The time for the rat to correct this posture was recorded and used an index of the intensity of catalepsy. Three trials were performed, with a brief rest in between. The average of all three trials was used for statistical analysis.

### Small animal DAT microPET imaging

For longitudinal in vivo measurement of DAT integrity, we used ^18^F-FE-PE2I, a highly selective DAT PET radioligand [37]. ^18^F-FE-PE2I produced under GMP was obtained from the hospital radiopharmacy at UZ Leuven.

Rats were anesthetized (5% isoflurane in O_2_ for induction and 2% thereafter at 1 L/min flow rate), placed on a heated mat and cannulated with a 23G catheter. They were then placed on the imaging bed (Molecubes, Ghent, Belgium) with integral temperature and respiration monitoring before being transferred to a β-cube microPET scanner (Molecubes/Bruker, Ghent, Belgium). Dynamic PET images were acquired for 90 min starting from intravenous injection with ^18^F-FE-PE2I (8.9 ± 2.7 MBq)/rat. Specific activity was 286 ± 119 GBq/micromol with mass doses injected between 0.1 and 0.4 nmol. Rats were kept under anesthesia during the entire procedure (2.5% isoflurane in O_2_ at 1 L/min flow rate), with temperature and respiration monitored throughout. After PET scanning, a computed tomography (CT) image was acquired for anatomic coregistration with an X-cube CT scanner (Molecubes, Ghent, Belgium), using the following parameters: 50kVp, 480 exposures, 85 ms/projection, 100 μA tube current, rotation time 60 s. After scanning, rats were recovered, with on average two weeks between scanning sessions.

#### Image processing and analysis

PET list mode data were binned into 16 frames (4 × 15 s, 4 × 1 min; /1 × 5 min /5 × 10 min and 2 × 15 min) and reconstructed into a 192 × 192 image matrix with 0.4 mm voxel size, using 30 iterations using the native Maximum-Likelihood Expectation–Maximization (MLEM) algorithm. Correction was done for randoms, scatter, attenuation based on CT, and data were scaled to kBq/cc after calibration to a standard ^18^F-phantom. Images were decay-corrected to the start of the scan. CT data were reconstructed using a regularized iterative algorithm [34] with a voxel size of 200 µm (isotropic) and were scaled to Hounsfield Units (HUs) after calibration against a standard air/water phantom. PET and CT data were then cropped to the skull and CT scans were co-registered to an in-house CT skull template using an affine transformation. The first 9 frames of PET data were summed for rigid co-registration with the CT scan. Dynamic PET data were then transformed to template space using the CT-based transformation matrices. Striatal and cerebellar regions of interest in template space [39] and time-activity curves were created (operations carried out using PFUS (https://store.bruker.com/products/pfus-image-fusion-remote/)). Time activity curves were imported in PKIN, where a standard reference tissue model (SRTM) was applied to generate non-displaceable binding potential (BPnd) values as measure for absolute tracer binding, using the cerebellum as the reference tissue [41]. Parametric BPnd images were then generated using the voxel SRTM method in PXMOD (PMOD Technologies GmbH, Zürich, Switzerland) with the same reference region.

### RNAscope in situ hybridization

For the staining of the brain (in situ hybridization and immunostaining), rats were sacrificed with an overdose of sodium pentobarbital (200 mg/kg, i.p., Dolethal, Vetoquinol, Lure, France), and transcardially perfused with saline and 4% paraformaldehyde (PFA) in PBS. After post-fixation overnight in 4% PFA, 20 µm thick coronal brain sections were made with a vibrating microtome (HM 650 V, Microm).

For RNAscope in situ hybridization, brain sections were transferred to a 48-well plate and washed once with PBS containing 0.1% Tween-20. Sections were then incubated with RNAscope™ Hydrogen Peroxide (Advanced Cell Diagnostics) for 10 min at room temperature (RT), protected from light. Tissue sections were mounted onto Superfrost™ Plus slides (Thermo Fisher Scientific), and were allowed to air-dry for 1 h at RT, then baked for 30 min at 60 °C to ensure proper adhesion of the tissue. Sections were then post-fixed for 1 h at 4 °C in 4% PFA in PBS. After fixation, sections were dehydrated using an ethanol gradient (50%, 70%, 100%, and 100%, 5 min each). The day after, target retrieval was performed using a steamer and 1X Target Retrieval Reagent solution (Advanced Cell Diagnostics) for 10 min. Next, protease digestion was performed using RNAscope Protease III reagent (Advanced Cell Diagnostics, V2 assay) for 30 min at 40 °C. For RNA detection, probe targeting *Atp10b* RNA from *Rattus norvegicus* (RNAscope Probe—Rn-Atp10b, catalog # 1155561-C1, Advanced Cell Diagnostics) was added to the brain sections. An additional section was incubated with RNAScope 3-plex Negative Control Probe (RNAscope 3-plex Negative Control Probe, catalog # 320871, Advanced Cell Diagnostics) which binds to *DapB*, a gene present in Bacillus subtilis strain. For signal amplification, RNAScope Multiplex FL v2 amplifiers (Advanced Cell Diagnostics) were added. Following probe hybridization and amplification steps, fluorescent signal detection was performed using the RNAscope Multiplex Fluorescent v2 Assay (Advanced Cell Diagnostics). For channel 1 (C1) development, RNAscope Multiplex FL v2 HRP-C1 reagent was applied to each section and incubated at 40 °C for 15 min. Slides were then washed twice in 1X RNAscope Wash Buffer for 2 min each at RT with gentle agitation. Tyramide signal amplification (TSA) was performed using TSA Plus Cyanine 5 (TSA 650, PerkinElmer), diluted 1:1500 in 1X RNAscope Multiplex TSA Diluent. Slides were incubated at 40 °C for 30 min. Following fluorophore development, slides were washed twice in 1X Wash Buffer (2 min each). To quench residual horseradish peroxidase (HRP) activity and prevent cross-reactivity in subsequent detection channels, RNAscope Multiplex FL v2 HRP Blocker was applied to each section and incubated for 15 min at 40 °C. Slides were then washed twice with 1X Wash Buffer for 2 min each at RT. RNAScope assay was combined with TH and RFP fluorescent staining. After the RNAscope protocol, we proceeded with fluorescent staining as explained in ‘Immunohistochemical staining’ Sect. below.

#### Analysis

RNAscope analysis was performed on either TH + or RFP + cells using ImageJ. Regions of interest (ROIs) were manually delineated by carefully outlining individual TH + or RFP + cells with clearly distinguishable nuclei that did not overlap with neighboring cells. The “Find Maxima” tool in ImageJ (Prominence > 10) was used to automatically detect and quantify Atp10b RNA puncta within each ROI. For the analysis of RFP + cells, the total number of Atp10b puncta detected in all the ROIs of one section was divided by the number of ROIs, resulting in an average puncta count per cell in one SN section.

### Immunohistochemical staining

Immunohistochemistry (chromogen) staining and immunofluorescence staining were performed on free-floating sections. Sections were washed with PBS, and incubated in an antigen retrieval solution (0.1 M citrate buffer pH 6.0) for 30 min at 80 °C. After 20 min on ice, the sections were washed in PBS, and incubated in 3% H_2_O_2_ and 10% methanol in PBS for 10 min at RT. The sections were washed 2 × 5 min in PBS with 0.1% Tergitol (PBS-T), and blocked with 10% goat serum, or 10% donkey serum depending on the species of the secondary antibodies used, for 1 h at RT. Then sections were incubated overnight in primary antibody diluted in PBS-T with serum at RT. The next day, the sections were washed 2 × 5 min in PBS-T, followed by biotinylated secondary antibody for 1 h at RT (for immunohistochemistry chromogen staining) or fluorescent secondary antibody for 2 h at RT (for immunofluorescence staining). Then, for immunohistochemistry chromogen staining, the sections were washed 2 × 5 min in PBS-T and incubated with streptavidin–horseradish peroxidase complex. Following 2 × 5 min washes in PBS-T, TH immunoreactivity was visualized using DAB 3,3’diaminobenzidine tetrahydrochloride. For immunofluorescence staining, after being rinsed in PBS and allowed to dry, the sections were coverslipped with Mowiol.

TH Immunofluorescence of the SNpc for stereological quantifications was performed on sections mounted on Superfrost plus glass slides, dried overnight and following an antibody signal enhancement (ASE) protocol [25]. Sections were pretreated with antigen retrieval solution (Tris–HCl-EDTA buffer pH 9.0 + 0.05% SDS) in the steamer for 30 min. After 20 min on ice, sections were washed with ASE wash buffer (PBS + 0.5% Tween-20) and blocked for 30 min with ASE blocking solution (PBS + 2% donkey serum, 50 mM glycine, 0.05% Tween-20, 0.1% Tergitol, 0.1% BSA). Sections were incubated overnight at 4 °C with primary antibody in ASE primary antibody buffer (PBS + 10 mM glycine, 0.05% Tween-20, 0.1% Tergitol, 0.1% H_2_O_2_). Next day, after one rinse and 2 × 3 min washes with ASE wash buffer, sections were incubated for 2 h at RT with secondary antibody diluted in ASE secondary buffer (PBS + 0.1% Tween-20). After being rinsed in PBS and allowed to dry, the sections were covered with Mowiol.

See Supplementary Table 1 for antibody information.

#### Analysis

Images of *dSTR* were captured with the Aperio CS2 slide scanner. QuPath software was employed to measure TH Optical Density (OD) in 6 sections per animal across the *dSTR* (sampling frequency of every twelfth sections, in a rostro-caudal manner).

The number of TH + /RFP + cells at 2 weeks post-injection (Fig. 1) and the number of TH + cells in the SNpc at 1 month and 1 year post-injection (Fig. 5) was determined by stereological measurements using the optical fractionator method in a computerized system as described before [2], in a blinded manner. The software used was Stereo Investigator (MicroBrightField, Delft, The Netherlands), and the hardware Leica Application Suite 3.6. The parameters were the following: frame area = 20 000 μm^2^, frame height = 10 μm, guard height = 2 μm, fame spacing = 300 μm, thickness = 17–19 μm, coefficient error = 0.09–0.17. For each animal, we analyzed 7 sections throughout the entire SNpc (sampling frequency of every tenth sections, in a rostro-caudal manner).

The number of TH + cells and HuC/D + cells in the SNpc (Fig. 6) was determined by automatic cell detection using QuPath software. For each animal, we analyzed 3–4 sections at different levels of the SN. Brain sections were imaged on a Zeiss Axioscan Z.1 slidescanner equipped with a Zeiss Colibri 7 illumination source and a Hamamatsu Orca Flash 4.0 V3 camera. Images were taken with a 10 × Plan-Apocromat objective (NA 0.45), at a sampling rate of 0.650 μm/pixel. The setup was controlled by ZEN blue (software version 3.8, Carl Zeiss Microscopy GmbH).

The number and volume of the late endosomes/lysosomes was determined in TH + dopaminergic neurons (Fig. 7) in 3 sections per animal spanning the SNpc. Images were captured using a Nikon AX confocal microscope at 60 × magnification. The captured Z-stacks were 3D reconstructed using Imaris version 10.0.1 (Oxford Instruments, Abingdon, United Kingdom), and labeled organelles were quantified using Imaris Spot detection within delineated TH + cell surfaces within the field of view.

### Protein extraction and Western blot

For protein expression analysis, rats were sacrificed with an overdose of sodium pentobarbital (200 mg/kg, i.p.), and transcardially perfused with saline. SN tissue was freshly isolated, snap-frozen and stored at – 80 °C until analysis. Samples were weighed and homogenized in 10 volumes of RIPA buffer (50 mM Tris–HCl, 150 mM NaCl, 0.1% (w/v) SDS, 1% (v/v) Triton-X100, 0.5% (w/v) Sodium Deoxycholate, 1.0 mM EDTA pH 7.4) containing a protease inhibitor cocktail (Roche complete) and phospho-STOP EASYPACK (Roche) using a tissue homogenizer (TH, Omni Tissue Homogenizer). After homogenization, samples were sonicated 3 times during 15 s, and centrifuged at 6000 g for 10 min at 4 °C. Protein sample concentration was determined by BCA protein assay (Thermo Scientific, MA, USA) according to the manufacturer’s directions.

Western blots were performed using 15 µg of SN extracts prepared in 4 × Laemmli buffer (0.24 M Tris pH 6.8, 7.27% SDS, 40% Glycerol, 10% β-Mercaptoethanol, 0.01% Bromophenol blue). Protein samples were loaded on 4–15% Criterion™ Tris–HCl Protein Gel and transferred to a polyvinylidene fluoride membrane (Bio-Rad). Nonspecific binding sites were blocked for 1 h at RT in 5% nonfat milk in PBS-T. After overnight incubation at 4 °C with primary antibodies, blots were washed 3 × 10 min with PBS-T and incubated with horseradish peroxidase-conjugated secondary antibody for 1 h. After 3 × 10 min with PBS-T, bands were visualised using Clarity Western ECL (Bio-Rad) and developed with a GE ImageQuant 800 (GE Healthcare). Densitometric analysis was performed using ImageQuant.

See Supplementary Table 1 for antibody information.

### Generation of isogenic knockout lines

The human induced pluripotent stem cells (iPSCs) BJ SiPS-D TH-TdTomato line was cultured using StemflexTM medium (ThermoFisher). Before nucleofection, the cells were pre-treated with a 10 μM Rhok inhibitor for an hour. The cells were then dissociated using Accutase, after which they were pelleted and resuspended in 800 μl PBS containing 5 μg of px330 CRISPR DNA each. Finally, they were transferred into nucleofector cuvettes. Nucleofection was performed using the P4 Nucleofector kit from Amaxa and the standard and program hiPSC CA-137. Two CRISPR sgRNA targeting exon1 were used to produce the knockout (KO) (CR1: CACCGCTACAACTTGACACAGCAG, AAACCTGCTGTGTCAAGTTGTAGC CR2: CACCGAATTGCTCAAAGAGATTCCG, AAACCGGAATCTCTTTGAGCAATTC), genotype primers used were F: TGGCAGTGGAGAGTCAGAGA, R: CCTGGGGAACAGAATGAGAC. Clones with homozygous or compound heterozygous deletions, leading to truncations and frameshift mutations, were identified using genotyping PCR. Clones for all lines containing deletions were identified by Sanger sequencing. Two generated ATP10B KO clones were used in this study, termed ATP10B KO clone#1 and ATP10B KO clone#2.

### Midbrain differentiation

Human iPSCs were cultured in StemflexTM medium (ThermoFisher) at 37 °C, with 5% CO_2_ in a humidified incubator, as previously described. Differentiation was done by dual-SMAD inhibition with SB431542 (R&D Systems, 10 μM), LDN193189 (Stemgent, 100 nM), B27 minus Vit A and N2 in DMEM-F12. Midbrain-specific patterning for midbrain NPCs was made with the addition of CHIR99021 (Stemgent, 3 μM), Purmorphamine (STEMCELL, 2 μM), and SAG (Abcam, 1 μM). Post patterning Neural maturation medium was DMEM F12 medium containing N2, B27-VitA, 20 ng/mL GDNF (R&D Systems), 20 ng/mL BDNF (R&D Systems), 0.2 mM ascorbic acid (Sigma), 0.1 mM dibutyryl cAMP (Biolong), 10 μM DAPT (Cayman Chemical). The medium for long-term culture was DMEM F12 medium containing N2, B27-VitA, 10 ng/ML GDNF (R&D Systems), 10 ng/mL BDNF (R&D Systems), 0.2 mM ascorbic acid (Sigma).

#### Staining and quantification

Cells were fixed with 4% PFA for 20 min, blocked in 0.1% Triton X-100 in 5% horse serum/PBS, and then incubated in primary antibodies overnight at 4 °C. The following day, cells were washed and incubated in secondary antibodies and DAPI nuclear stain according to protocol. Imaging was performed using the High-content imager CX7 with phenotyping and quantifications using CellProfiler. The average of at least three fields of a well was counted as the N value for statistical analysis.

See Supplementary Table 1 for antibody information.

#### Time-course FACS analysis

Midbrain neuronal cultures or iPSC cultures were dissociated at different time points with Accutase, resuspended in PBS supplemented with 1xB27, DNase, and 10 μM Y-27632 ROCK inhibitor, and filtered through a 35 μm strainer into a 5-ml Falcon round-bottom tube. The BD Celesta analyzer (BD Biosciences) was used to measure TH-TdTomato-positive cells. The flow cytometry results were analyzed using FlowJo™ v10.8 Software (BD Life Sciences).

#### Live-cell caspase-3/7 activity assay

Active caspase-3/7 activity was detected using the LIVE/DEAD™ Image-iT™ Caspase-3/7 Detection Kit (Thermo Fisher Scientific) according to the manufacturer’s instructions. Briefly, iPSC-derived neuronal cultures were incubated with the caspase-3/7 detection reagent at 37 °C for 1 h to label cells undergoing apoptosis. Following live staining, cells were immediately fixed with 4% paraformaldehyde for 15 min at room temperature. Fixed cells were then subjected to immunofluorescence staining.

### Statistical analysis

Data are presented as mean ± standard error of the mean (s.e.m.), and individual values. Statistical analyses were performed using GraphPad Prism version 10.2.0. For analyzing paired observations within one group of animals, we used a parametric paired two-tailed t-test or non-parametric Wilcoxon signed-rank test. For all the other analyses, we employed either a one-way ANOVA, or two-way ANOVA, followed by post-hoc comparisons. Correlation analyses were performed using Pearson correlation. Parametric or non-parametric statistical tests were performed based on the normality of residuals and equal variance across groups, tested using the D’Agostino-Pearson omnibus and Brown-Forsythe tests respectively. The α-value was set at 0.05.

## Results

### In vivo KD of ATP10B in adult rat SNpc by stereotactic injection of AAV vectors

*ATP10B* has recently emerged as a candidate genetic risk factor in the context of PD. Studies have identified loss-of-function mutations and reduced *ATP10B* mRNA expression in PD patients [16]. To gain a deeper understanding of ATP10B’s role in PD pathogenesis, and specifically in the nigrostriatal dopaminergic system, we conducted a study employing a rat model with ATP10B KD targeted to the SNpc neurons (Fig. 1a). To knock down ATP10B, we used two different miRNA-based shRNA sequences (miR5 and miR7) that bind to two different regions of the *Atp10b* mRNA (Fig. 1b). We previously showed that these sequences induce loss of ATP10B expression and sensitize primary mouse cortical neuron cultures to PD-related stressors [16]. A scrambled sequence (SCR) was used as control. Two weeks post-injection, we assessed the transduction efficiency and construct expression of the AAV2/7 miR5, miR7, and SCR vectors in SNpc dopaminergic neurons based on RFP expression. Our findings revealed that all three vectors performed similarly, demonstrating high transduction rates of TH + cells (~ 90%) (Fig. 1c, d).

To measure *Atp10b* RNA levels in targeted SNpc neurons at 1 year post-injection, we performed RNAscope in situ hybridization in combination with TH and RFP immunostaining. First, we confirmed the presence of *Atp10b* RNA in dopaminergic neurons, by showing a significantly higher number of *Atp10b* RNA puncta per TH + cell compared to the negative probe (Fig. S1a, b). Next, we quantified the number of *Atp10b* RNA puncta in RFP + cells within the SNpc across the different injected groups. *Atp10b* RNA levels were significantly reduced in miR7 transduced cells compared to SCR (miR7 vs. SCR: *p* = 0.0095), suggesting a more robust KD effect in miR7 compared to miR5 (Fig. S1c, d).

### ATP10B KD in SNpc neurons leads to decreased motor function and induces motor asymmetry

**Fig. 2:**
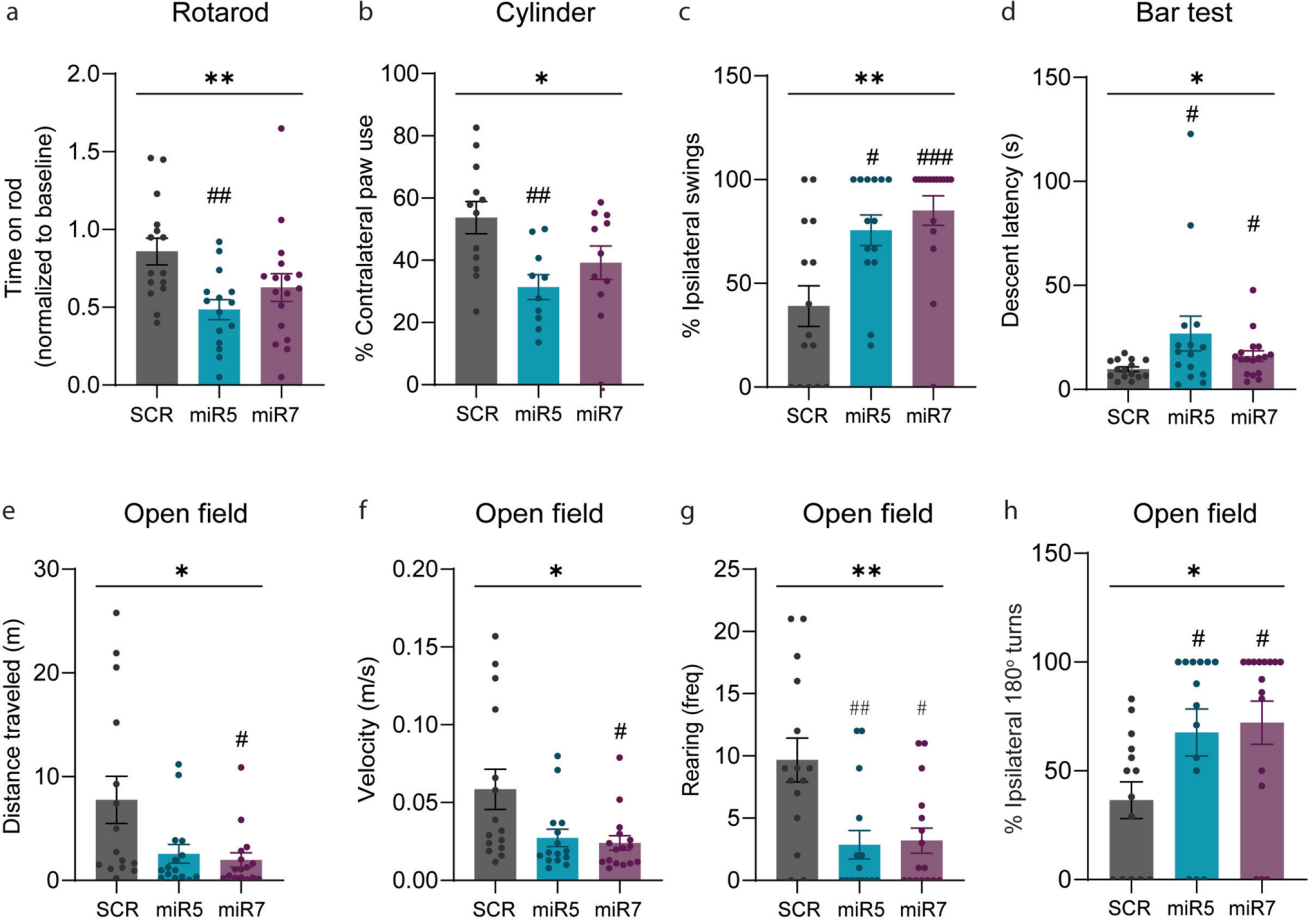
ATP10B KD in SNpc neurons leads to parkinsonian motor deficits at 12 months post injection. **a** Motor coordination and balance was assessed using an accelerated rotarod test (4–40 rpm), with the average performance post-lesion normalized to the individual rat performance prior to surgery. **b**,** c**,** h** Motor asymmetry was examined by quantifying the preference in the use of the contralateral paw in the cylinder test, the bias to ipsilateral swing in the EBST, and the spontaneous ipsilateral turning behavior in the open field test. **e**,** f**,** g** Spontaneous motor behavior was recorded during a 5-min open field test and analyzed for total distance traveled, velocity, and frequency of rearing. **d** The catalepsy bar test was performed to assess muscular rigidity. Time spent by the rat grabbing the elevated bar before correcting its posture on the floor was measured. In (**a**, **c–h**) data are mean ± s.e.m, and analyzed using non-parametric one-way ANOVA (Kruskal Wallis) (*p < 0.05, **p < 0.01) and Dunn’s post-hoc test versus SCR (#*p* < 0.05, ##*p* < 0.01, ###*p* < 0.001). SCR (*n* = 15), miR5 (*n* = 15), miR7 (*n* = 17). In (**b**) data are mean ± s.e.m and analyzed using one-way ANOVA (*p < 0.05) and Dunnett’s post-hoc test versus SCR (##*p* < 0.01). SCR (*n* = 12), miR5 (*n* = 10), miR7 (*n* = 11). Each dot represents the value of one animal

To evaluate the motor performance of the ATP10B KD rats, we conducted a series of behavioral tests at different times post injection. We will focus on the behavioral test results at 1 year, as this time point was chosen for the neuropathological examination of the brain, although motor impairments could be observed from earlier time points post injection (Fig. S2). In the rotarod test, no significant differences could be observed between the groups at baseline, indicating equal acquisition of rotarod motor skill (data not shown). At 12 months post-injection, we noted an overall compromised motor coordination and balance, as evidenced by the time spent on top of the rod across the three groups (treatment factor: *p* = 0.0083). Post hoc analysis revealed that a decreased time on top of the rod was observed in the miR5 group compared to the SCR (miR5 vs. SCR: *p* = 0.0043) (Fig. 2a). An overall treatment effect was also evidenced in the cylinder test, where the percentage of left (non-injected) forepaw usage was quantified (treatment factor: *p* = 0.0110). Post hoc analysis revealed that miR5 group had a significant decrease in left forepaw usage when compared to SCR (miR5 vs. SCR: *p* = 0.0068) (Fig. 2b). This motor asymmetry observed in the cylinder test was consistent with the findings in the EBST (Fig. 2c) and the turning behavior in the open field (Fig. 2h). Indeed, both the miR5 and miR7 groups exhibited a strong preference for performing ipsilateral body swings in the EBST (treatment factor: *p* = 0.0016; miR5 vs. SCR: *p* = 0.0321, miR7 vs. SCR: *p* = 0.0009) (Fig. 2c) and spontaneous ipsilateral rotations in the open field (treatment factor: *p* = 0.0123; miR5 vs. SCR: *p* = 0.0388, miR7 vs. SCR: *p* = 0.0112) (Fig. 2h) compared to the SCR group. In addition, overall impaired locomotor activity and exploration behavior was also observed in the open field, indicated by parameters such as distance traveled (treatment factor: *p* = 0.0433), velocity (treatment factor: *p* = 0.0487) and frequency of rearing (treatment factor: *p* = 0.0064) (Fig. 2e–g). While post hoc analysis revealed that the miR7 group exhibited a more pronounced impairment in distance traveled (miR7 vs. SCR: *p* = 0.0312) and velocity (miR7 vs. SCR: *p* = 0.0212), both miR5 and miR7 groups decreased their rearing behavior in the open field (miR5 vs. SCR: *p* = 0.083, miR7 vs. SCR: *p* = 0.0166). Notably, the catalepsy test evidenced an overall increased muscular rigidity (treatment factor: *p* = 0.0358), with a stronger effect observed in the miR5 group compared to SCR control group (miR5 vs. SCR: *p* = 0.0181). This was indicated by a more prolonged duration of holding the elevated bar before correcting their posture to the floor (Fig. 2d). In summary, ATP10B KD in SNpc neurons using 2 different short hairpin sequences leads to a spectrum of behavioral impairments, encompassing motor asymmetry and dysfunction, similar to those observed in PD patients.

### ATP10B KD in SNpc neurons results in a progressive loss of striatal DAT assessed by PET imaging

**Fig. 3:**
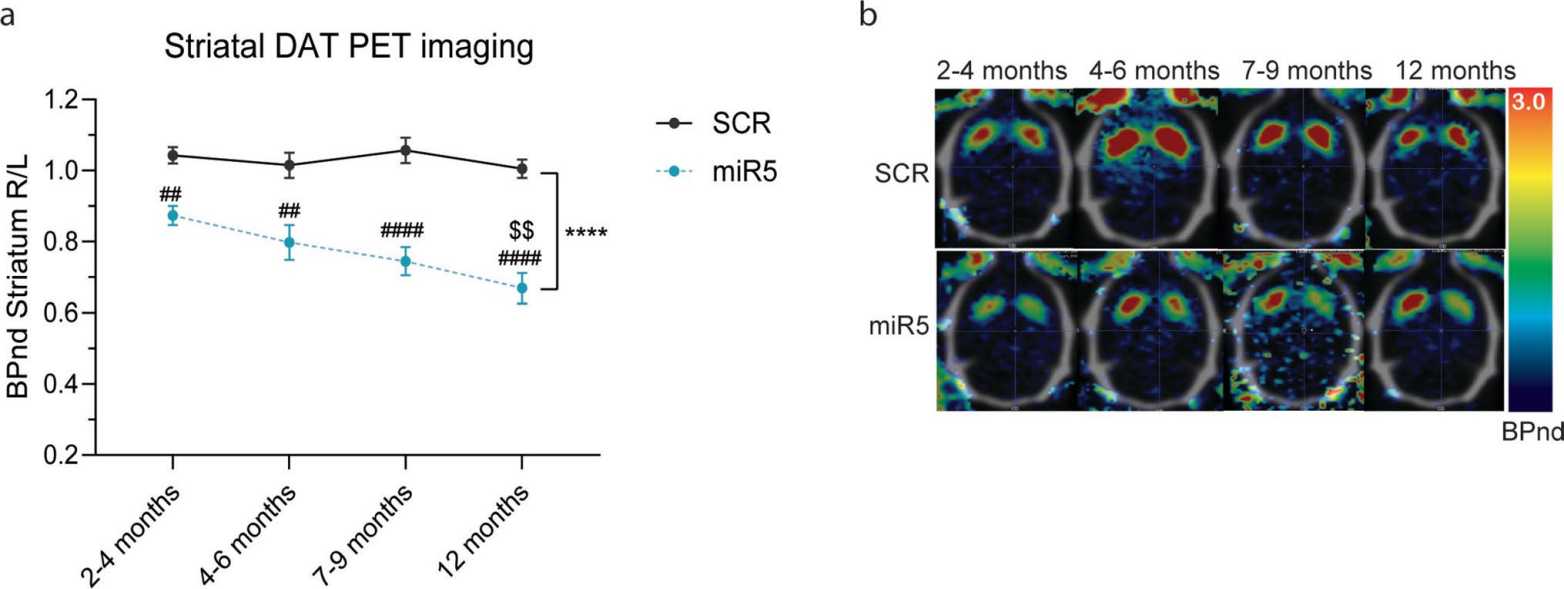
ATP10B KD in SNpc neurons leads to decreased striatal DAT binding. **a** Striatal DAT binding potential (BPnd), measured by ^18^F-FE-PE2I microPET imaging, in the ipsilateral (R) vs. the contralateral (L) striatum in miR5-injected animals (*n* = 5) and SCR-injected animals (*n* = 3) at different time points. Data are mean ± s.e.m. and analyzed using two-way ANOVA (****p < 0.0001 treatment factor) and Tukey post hoc tests contrasting miR5 vs. SCR at each corresponding time point (miR5 vs. SCR: ##*p* < 0.01, ####*p* < 0.0001), and treatment groups across time (miR5 12 months vs. miR5 2–4 months: $$*p* < 0.01). **b** Parametric images of ^18^F-FE-PE2I BPnd in representative SCR (upper) and miR5 (middle) rats over time. Transverse images are shown in neurological orientation and are overlaid onto a reference CT skull template with the miR5 lesioned striatum on the right-hand side

Longitudinal imaging with ^18^F-FE-PE2I was done on miR5 and SCR rats at four distinct time points: 2–4 months, 4–6 months, 7–9 months, and 12 months post-injection. The results are expressed as right versus left BPnd of ^18^F-FE-PE2I in the striatum of miR5 and SCR rats. BPnd values obtained from both hemispheres for the different groups and the different time points are shown in the Fig. S3. While SCR rats consistently exhibited a stable BPnd on both sides of the striatum over time, ATP10B KD miR5 rats displayed a reduction in ^18^F-FE-PE2I BPnd on the ipsilateral side (right) compared to the contralateral side (left) when compared to SCR (Fig. 3a, b). Additionally, the right versus left BPnd value in this group decreased progressively over time (2–4 months: ~ 13% *p* = 0.013, 4–6 months: ~ 21% *p* = 0.045, 7–9 months: ~ 26% *p* = 0.0055, 12 months: ~ 34% *p* = 0.0024), indicating a gradual loss of DAT in the presynaptic terminals of dopaminergic neurons of miR5 injected rats (miR5 2–4 months vs. 12 months: *p* = 0.0024). Similar directions of change were observed in a smaller sample of ATP10B KD miR7 rats imaged at 7–9 months (*n* = 2) (~ 66%) and 12 months (*n* = 3) (~ 53%) post injection (Fig. S3).

### ATP10B KD in SNpc neurons results in a progressive decrease of striatal dopaminergic terminals

**Fig. 4:**
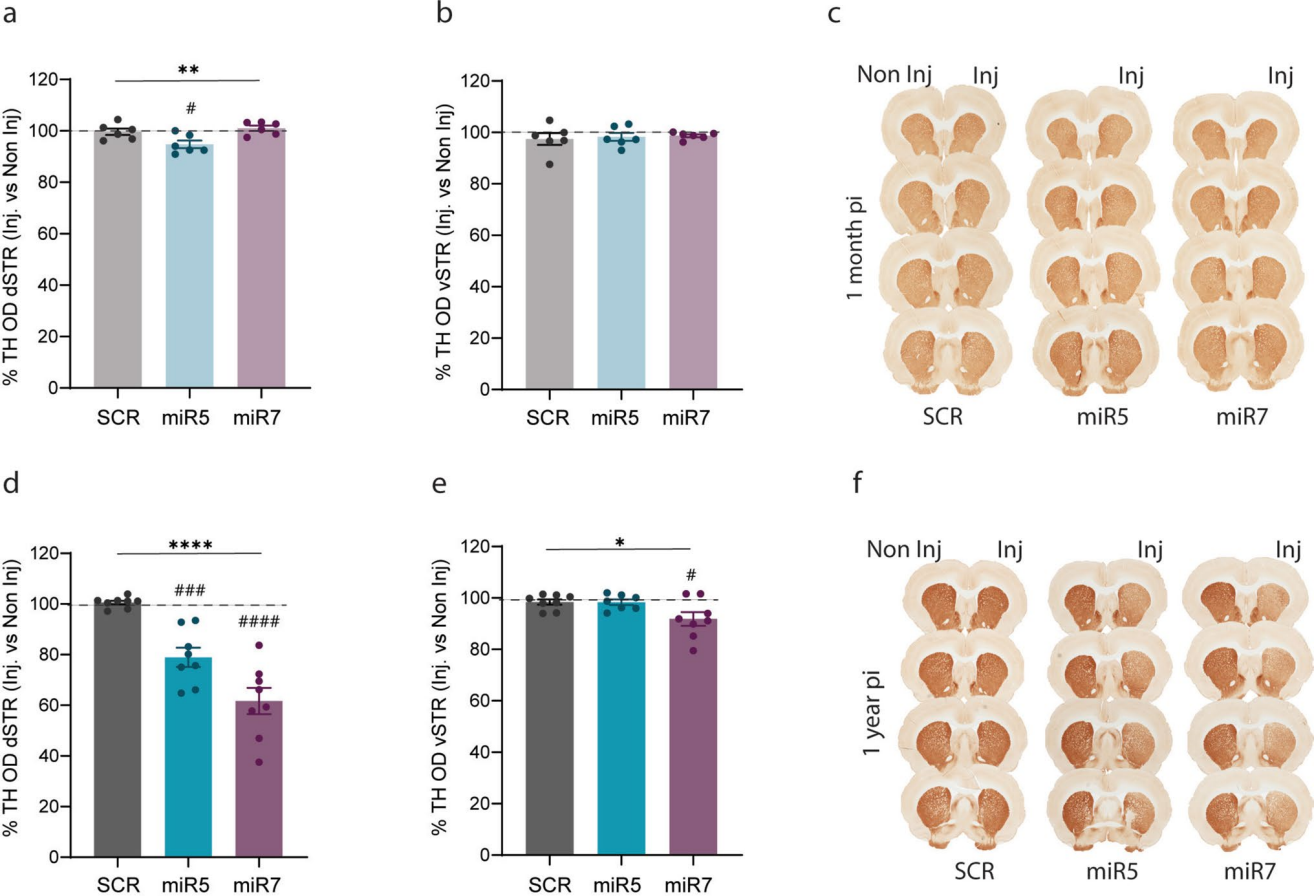
ATP10B KD in SNpc neurons leads to time-dependent loss of dopaminergic terminals in the dSTR. **a**,** b** Percentage of TH OD quantified in the injected versus the non-injected side in the dSTR (**a**) and vSTR (**b**) of 1 month post-injection rats. SCR (*n* = 4), miR5 (*n* = 5), miR7 (*n* = 6). **c** Representative images of TH immunohistochemical staining on 4 different sections from one SCR, miR5 and miR7 rat 1 month post-injection. **d**,** e** Percentage of TH OD quantified in the injected versus the non-injected side in the dSTR (**d**) and vSTR (**e**) of 1 year post-injection rats. SCR (*n* = 8), miR5 (*n* = 8), miR7 (*n* = 8). **f** Representative images of TH immunohistochemical staining on 4 different sections from one SCR, miR5 and miR7 rat 1 year post-injection. **a-b, d-e** Data are mean ± s.e.m, and analyzed using one-way ANOVA (*p < 0.05, **p < 0.01, ****p < 0.0001 treatment factor) followed Dunnett’s multiple comparisons test (#*p* < 0.05, ###p < 0.001, ####*p* < 0.0001 vs. SCR). Each dot represents the average of the 7 sections per animal

Overall, no loss of dopaminergic terminals was observed in the striatum at 1 month post- injections. We only observed a significant yet subtle decrease (~ 8%) in dopaminergic terminals in the miR5 group, as indicated by decreased TH OD in the dSTR when compared to SCR (miR5 vs. SCR: *p* = 0.0264) (Fig. 4a, c). At 1 year post-injection, there was a significant decline in dopaminergic terminals in the dSTR of both miR5 (~ 20%) and miR7 (~ 40%) rats, which was not present in the SCR group (miR5 vs. SCR: *p* = 0.0010, miR7 vs. SCR: *p* < 0.0001) (Fig. 4d, f). An initial pilot study in a separate cohort of rats injected with AAV2/7 SCR and miR5 vectors showed comparable results at 1 year post-injection, confirming the impact of ATP10B on dopaminergic terminal loss (Fig. S4). In contrast, at 1 year post-injection, the ventral striatum (vSTR) of the rats was less affected. Only the miR7 group showed a mild reduction in TH OD (~ 5–10%) at 1 year post-injection, which was statistically significant compared to SCR (miR7 vs, SCR: *p* = 0.0317) (Fig. 4e, f). No effect was observed at 1 month post-injection (Fig. 4b, c). Individual OD values of the injected and non-injected sides across all groups are presented in Fig. S5a, b (1 month) and Fig. S5d, e (1 year).

In conclusion, ATP10B KD in nigral neurons of rats results in a gradual loss of dopaminergic terminals in the dSTR.

### ATP10B KD in SNpc neurons results in a progressive loss of nigral dopaminergic neurons

**Fig. 5:**
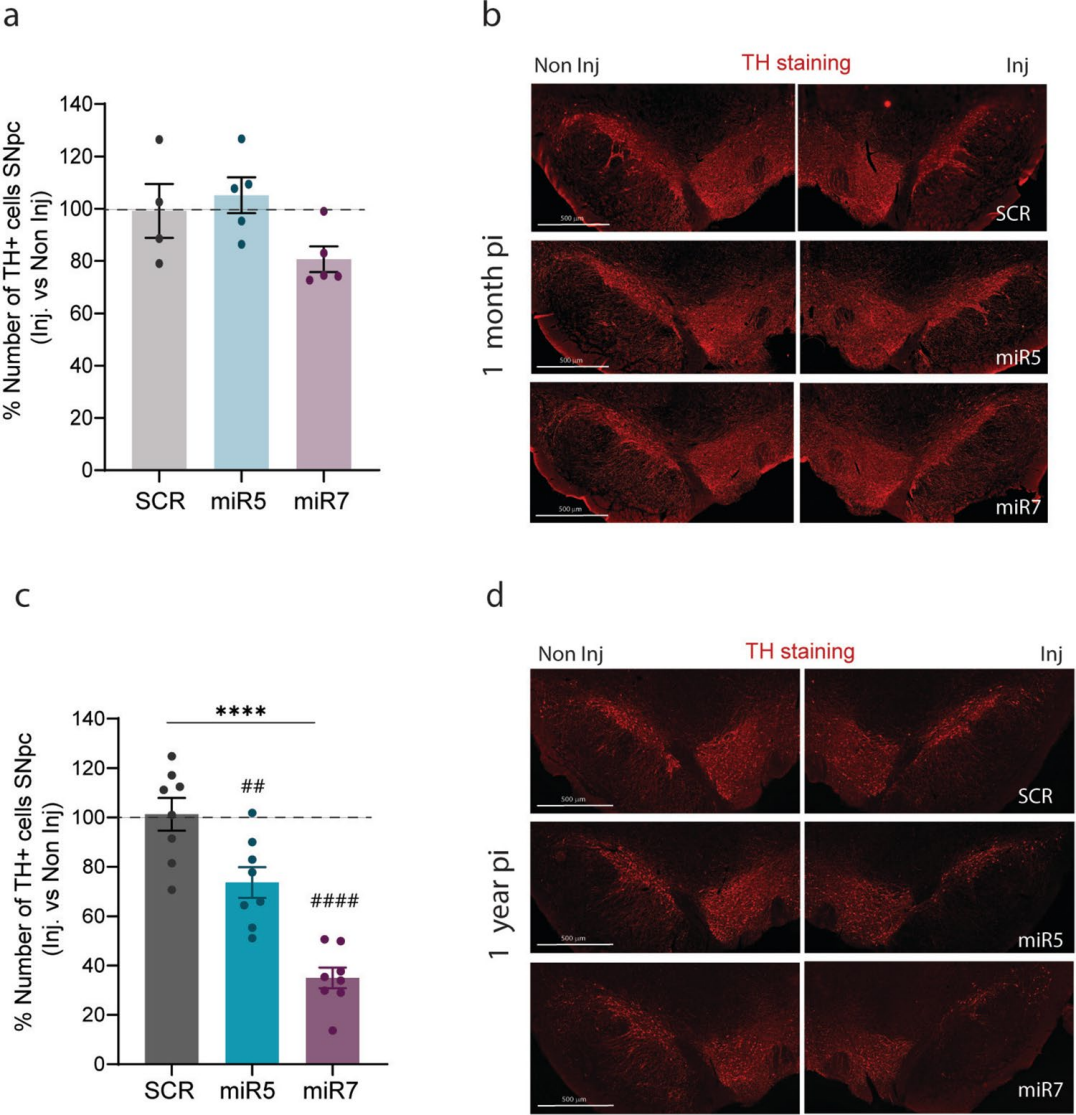
ATP10B KD in SNpc neurons leads to time-dependent loss of dopaminergic neurons. **a** Percentage of TH + cells quantified in the injected versus non-injected side in the SNpc of 1 month post-injection rats using stereology. SCR (*n* = 4), miR5 (*n* = 5), miR7 (*n* = 5). **b** Representative images of TH immunofluorescence staining on SNpc section from one SCR, miR5 and miR7 rat at 1 month post-injection. **c** Percentage of TH + cells quantified in the injected versus non-injected side in the SNpc of 1 year post-injection rats using stereology. SCR (*n* = 8), miR5 (*n* = 8), miR7 (*n* = 8). **d** Representative images of TH immunofluorescence staining on SNpc section from one SCR, miR5 and miR7 rat 1 year post-injection. **a**,** c** Data are mean ± s.e.m and analyzed using one-way ANOVA (****p < 0.0001 treatment factor) followed Dunnett’s multiple comparisons test (##*p* < 0.01, #### *p* < 0.0001 vs. SCR). Each dot represents the average of the 7 sections per animal

At 1 month post-injection, no significant loss of dopaminergic neurons was observed in the SNpc of either miR5 or miR7 injected rats when comparing to the SCR group (Fig. 5a, b). However, at 1 year post injection, both miR5 and miR7 injected rats exhibited a significant reduction in the number of TH + dopaminergic neurons in the SNpc (miR5 vs. SCR: ~ 25% *p* = 0.0053, miR7 vs. SCR: ~ 70% *p* < 0.0001) (Fig. 5c, d). Individual stereological counts of TH + cells of the injected and non-injected sides across all groups are presented in Fig. S5c (1 month) and Fig. S5f (1 year).

**Fig. 6:**
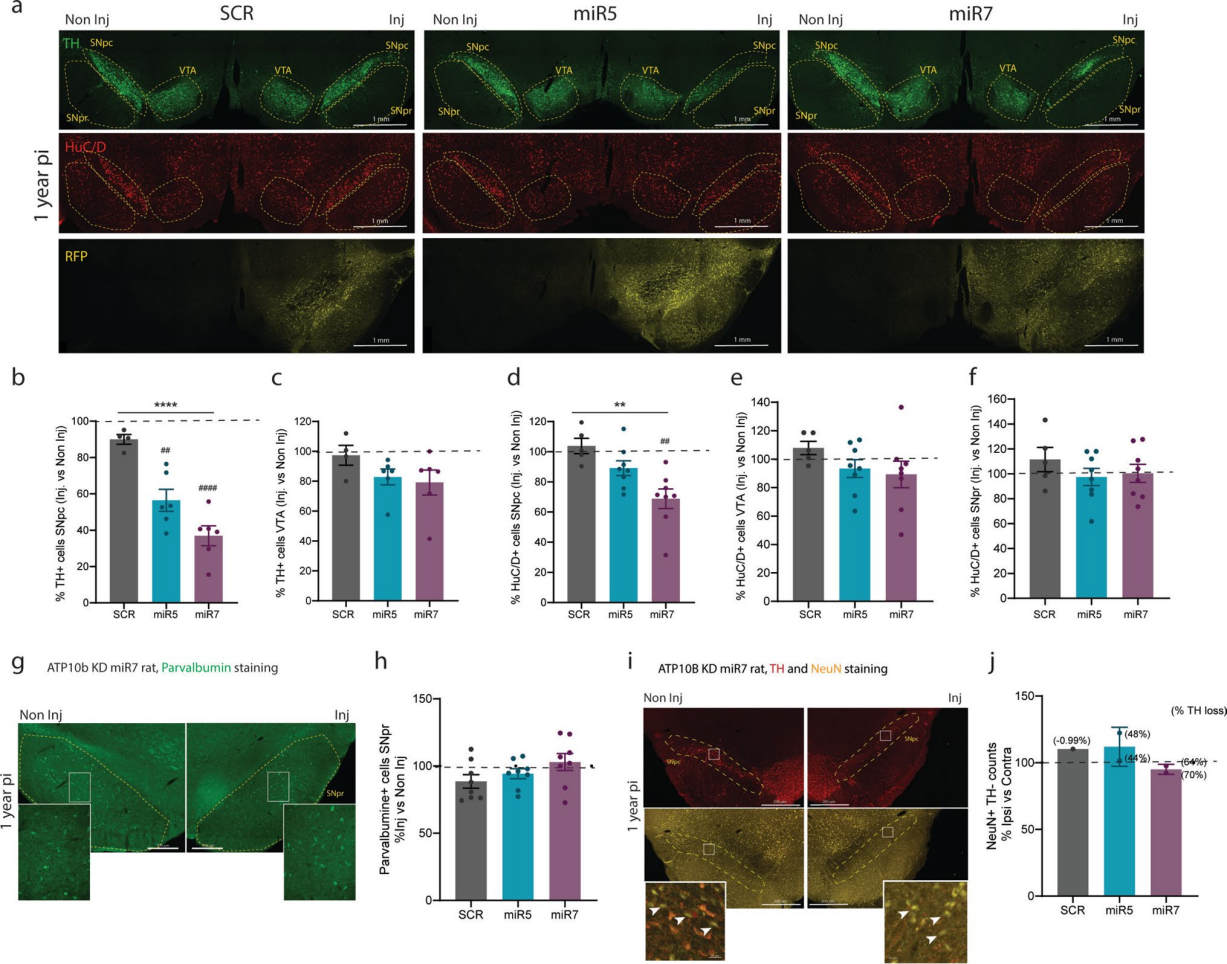
Analysis of dopaminergic and non-dopaminergic neurons in the SNpc, VTA and SNpr at 1 year post-injection**.** **a** Representative images of 1 year injected SCR, miR5 and miR7 rats stained with TH (green), HuC/D (red) and RFP (yellow) antibodies. Regions of interest are outlined with a dashed yellow line. **b, c** Percentage of TH + cells in the SNpc (**b**) and VTA (**c**) quantified in the injected versus non-injected side using QuPath. SCR (*n* = 4), miR5 (*n* = 6), miR7 (*n* = 6). **d-f** Percentage of Huc/D + cells in the SNpc (**d**), VTA (**e**) and SNpr (**f**) quantified in the injected versus non-injected side using QuPath. SCR (*n* = 5), miR5 (*n* = 8), miR7 (*n* = 8). **b-f** Each dot represents the average of 3–4 sections from one animal. **g** Representative immunofluorescent staining of parvalbumin in a 1 year injected miR7 rat. **h** Number of parvalbumin + cells in the SNpr showed as percentage in the injected versus the non-injected side of 1 year post-injection rats. Cells were counted using stereology in 7 sections that cover the SNpr. SCR (*n* = 1), miR5 (*n* = 3), miR7 (*n* = 3). Each dot represents one animal. **i** Representative images of TH and NeuN co-staining in a 1 year injected miR7 rat. Arrows indicate NeuN + TH- cells at both sides. **j** Number of NeuN + TH- cells in the SNpc showed as percentage in the injected versus the non-injected side. Cells were counted using ImageJ and individual data points indicate the average of three sections per rat. SCR *n* = 1, miR5 *n* = 2, miR7 *n* = 2. The percentage of TH + cell loss in the SNpc at 1 year post-injection in the corresponding rats is displayed between brackets. **b-f, h, j** Data are mean ± s.e.m and analyzed using one-way ANOVA (**p < 0.01, ****p < 0.0001 treatment factor) followed Dunnett’s multiple comparisons test (##*p* < 0.01, ####*p* < 0.0001 vs. SCR)

In addition, combined immunofluorescence staining of TH and the pan-neuronal marker HuC/D (Fig. 6a), revealed that dopaminergic neuron loss in the *SNpc* was accompanied by a reduction of HuC/D + neurons. This decrease was significantly more pronounced in the miR7 injected rats when compared to SCR (miR7 vs. SCR: *p* = 0.0015) (Fig. 6b, d). In a small subset of animals analyzed 1 year post-injection, quantification of TH-/NeuN + neurons showed similar non-dopaminergic neuronal populations in the SNpc for both miR5 and miR7 rats, despite the observed loss of TH + neurons (Fig. 6i, j). No loss of dopaminergic (TH +) or total neurons (HuC/D +) was detected in the ventral tegmental area (VTA) (Fig. 6c, e). We also assessed the total number of neurons (HuC/D +) and parvalbumin-positive GABAergic neurons in the substantia nigra pars reticulata (SNpr). No loss of HuC/D + (Fig. 6f) or parvalbumin + (Fig. 6g, h) neurons was found in miR5 or miR7 at 1 year post-injection.

In conclusion, these findings indicate that ATP10B depletion in SNpc neurons of adult rats leads to a progressive and selective loss of dopaminergic neurons in the SNpc, while neurons in adjacent regions such as the VTA and SNpr remain unaffected.

### ATP10B KD in SNpc neurons results in altered expression of endolysosomal markers in nigral dopaminergic neurons

Previous in vitro studies have demonstrated that loss of ATP10B has an impact on lysosomal activity. To investigate this further in our model, we first performed western blot analysis to assess the total levels of various lysosomal proteins in whole SN extracts obtained from miR5, miR7 and SCR groups at 1 year post-surgery. Specifically, we quantified the expression of LAMP1, GCase and Cathepsin B, as well as the autophagy receptor p62. Given the importance of lysosomes and autophagy in the clearance and degradation of α-synuclein, we also measured total α-synuclein levels. However, no global differences were observed between ATP10B KD and SCR groups for any of these proteins in total SN extracts (Fig. S6).

**Fig. 7:**
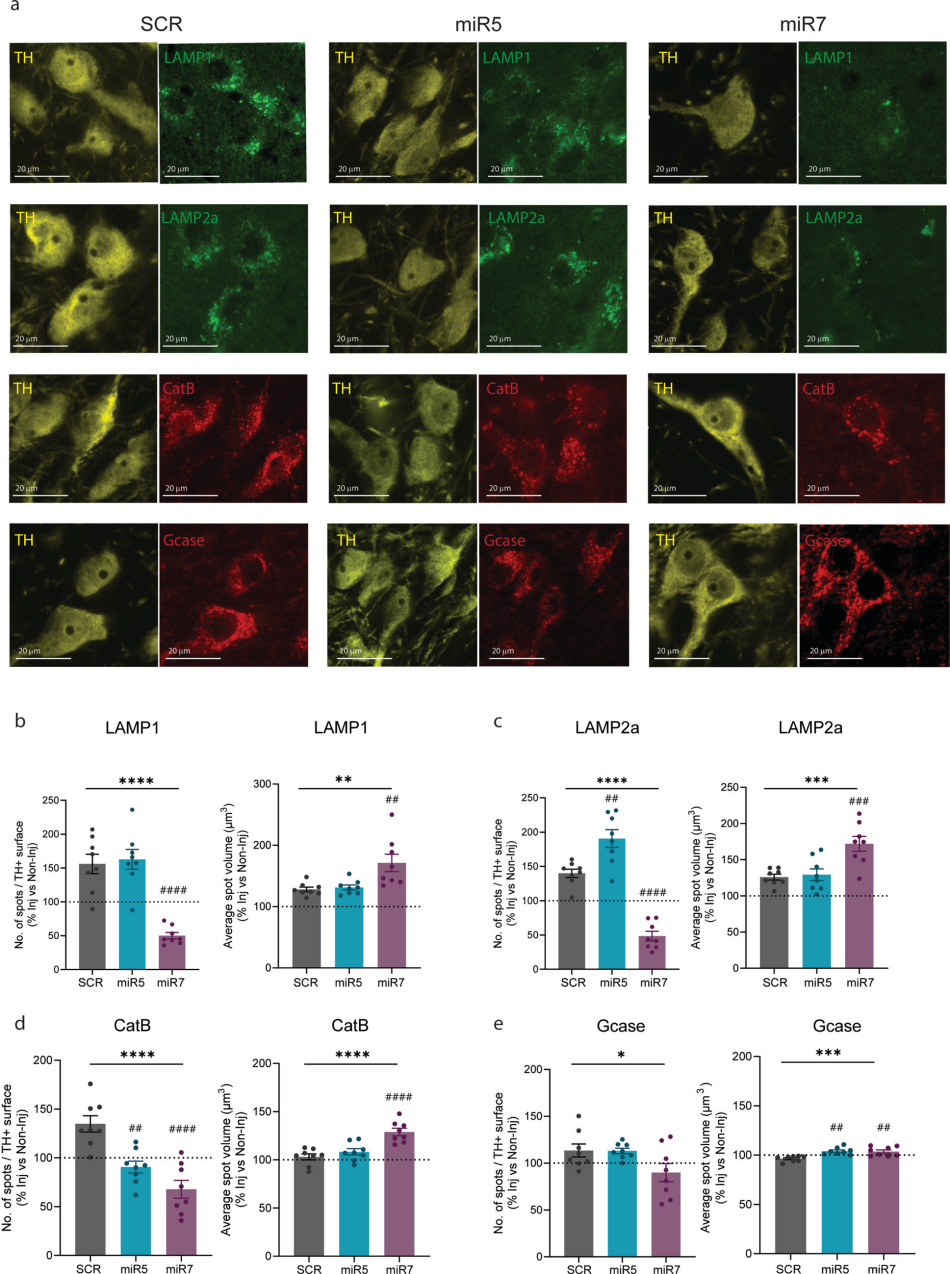
Immunofluorescent staining of late endosomal/lysosomal markers in dopaminergic neurons at 1 year post-injection. **a** Representative images of LAMP1, LAMP2a, cathepsin B and GCase co-stained with TH marker in the SNpc of SCR, miR5 and miR7 rats at 1 year post-injection. **b-e** Density of LAMP1 + , LAMP2a + , cathepsin B + , or GCase + spots (number of spots per total TH + surface) and average spot volume in TH + cells quantified in the injected side versus non-injected side using Imaris ver. 10.0.1. SCR (*n* = 8), miR5 (*n* = 8), miR7 (*n* = 8). **b** LAMP1 + number of spots and LAMP1 + average spot volume. **c** LAMP2a + number of spots and LAMP2a + average spot volume. **d** Cathepsin B + number of spots and cathepsin B + average spot volume. **e** GCase + number of spots and GCase + average spot volume. Data are mean ± s.e.m and analyzed using one-way ANOVA (*p < 0.05, ***p < 0.001, ****p < 0.0001 treatment factor) followed Dunnett’s multiple comparisons test (##p < 0.01, ###*p* < 0.001, ####*p* < 0.0001 vs. SCR), with the exception of LAMP1 average spot volume which was analyzed using Kruskal–Wallis (**p < 0.01, treatment factor) followed Dunn’s multiple comparisons test (##*p* < 0.01 vs. SCR). Each dot represents the average of 3 sections per animal

Next, we wanted to further explore lysosomal changes that may occur specifically in SNpc dopaminergic neurons and might be masked in whole tissue extracts. Consequently, we conducted immunofluorescence staining of different late endosomal/lysosomal markers together with TH in the SNpc of the ATP10B KD rats (Fig. 7a). Interestingly, the miR7 group presented a decrease in the number of LAMP1 + and LAMP2a + late endosomes/lysosomes in dopaminergic neurons compared to SCR (LAMP1 miR7 vs. SCR: *p* < 0.0001, LAMP2a miR7 vs. SCR: *p* < 0.0001). Additionally, the LAMP1 + and LAMP2a + late endosomes/lysosomes in the miR7-injected animals displayed an enlarged volume (LAMP1 miR7 vs. SCR: *p* = 0.006, LAMP2a miR7 vs. SCR: *p* = 0.0009) (Fig. 7b, c). When examining the expression of the lysosomal hydrolase cathepsin B, a reduction was noted in the number of cathepsin B + organelles in the dopaminergic neurons of both the miR7 and miR5 groups compared to SCR (miR7 vs. SCR: *p* < 0.0001, miR5 vs. SCR: *p* = 0.0016). Notably, these lysosomes also presented an enlarged volume, an effect particularly noticeable in miR7-injected rats (miR7 vs. SCR: *p* < 0.0001) (Fig. 7d). Although no alterations were detected in the number of GCase + organelles in any of the KD groups, a slight increase in GCase + lysosomal volume was discerned in both the miR5 and miR7 groups compared to SCR (miR5 vs. SCR: p = 0.0012, miR7 vs. SCR: *p* = 0.0020) (Fig. 7e). It is worth noting that rats injected with the control SCR vector showed an increase in the number of late endosomes/lysosomes in the dopaminergic neurons of the injected side compared to the non-injected side (Fig. 7b–e), suggesting an effect attributed to the injection of a viral vector containing a shRNA sequence and RFP expression. Representative images and quantification from both injected and non-injected side of the lysosomal markers are presented in Fig. S7.

In summary, our findings highlight the sensitivity of dopaminergic neurons to reduced ATP10B levels in rats. Knocking down ATP10B in the neurons of the SNpc leads to progressive neurodegeneration of dopaminergic neurons within this brain region, mirroring the loss of TH + terminals in the dSTR. The dopaminergic neurons in the rats injected with both ATP10B KD vectors showed a decrease in the number of cathepsin B + organelles compared to SCR. In addition, in the group of rats injected with miR7 we could observe a reduction in the number of late endosomes/lysosomes (marked by either LAMP1, LAMP2a, or cathepsin B) together with an enlargement in the volume of these organelles in dopaminergic neurons.

### ATP10B KO results in a decreased number of dopaminergic neurons in human iPSC-derived midbrain culture

**Fig. 8:**
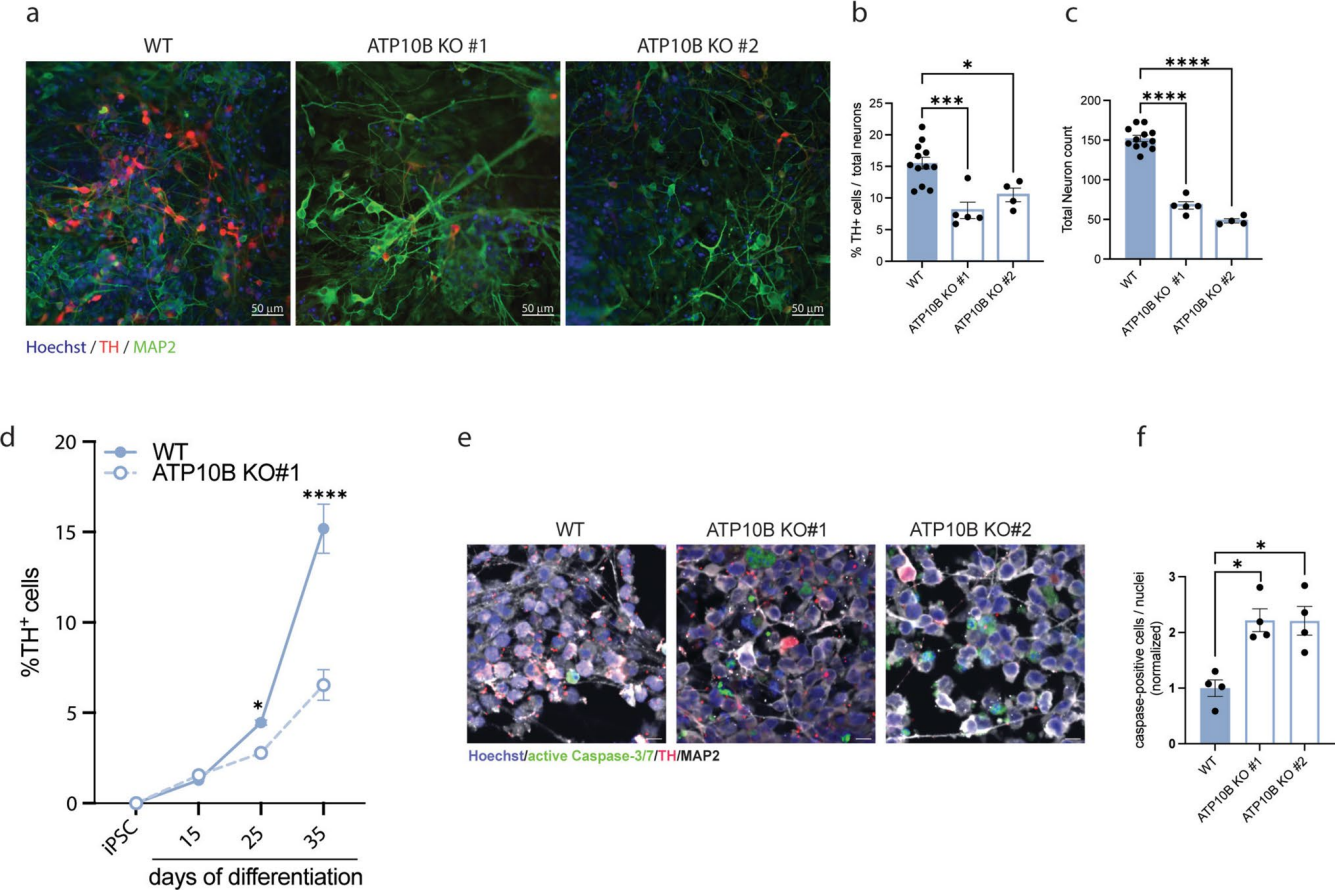
ATP10B KO results in decreased number of dopaminergic neurons in human iPSC-derived midbrain culture. **a** Immunocytochemistry pictures of midbrain neuronal cultures stained for TH (red) MAP2 (green) and Hoechst (blue). Scale bar 50 µm. **b, c** Quantification of neuronal populations: percentage of TH + cells over total MAP2 + population (**b**) and total number of MAP2 + cells (**c**) in WT and ATP10B KO clone#1 and clone#2. **d** FACS analysis of the TH-positive population in WT and ATP10B KO clone#1in a time course experiment. **E** Immunofluorescence staining of midbrain neuronal cultures at day 25 for active caspase-3/7 (green), TH (red) and MAP2 (grey) and **f** quantification of caspase-positive cells / nuclear counts, normalized to the WT condition. **b**,** c**,** f** Data are mean ± s.e.m and analyzed using one-way ANOVA followed by Dunnett’s multiple comparison test. Each data point is the average of 3 fields of view from one well. **d** Data are mean ± s.e.m. and analyzed using two-way ANOVA and Tukey post hoc test ATP10B KO #1 vs. WT at corresponding time point (*p < 0.05, ***p < 0.001, ****p < 0.0001)

To validate the susceptibility of dopaminergic neurons to the loss of ATP10B in a more translational model, ATP10B KO cell lines were generated in a TH-TdTomato reporter human iPSC line (BJ-SiPS) with genetic isogenic background (Fig. S8a–b). Cells were differentiated into midbrain neurons following a previously published protocol [14]. On day 30 of differentiation, we evaluated the number of dopaminergic and total neurons generated. We found a significant reduction in the number of TH-positive (WT vs. clone#1 *p* = 0.0005, WT vs. clone#2 *p* = 0.023) (Fig. 8a, b) and MAP2-positive (*p* < 0.0001 for clone#1–2) neurons (Fig. 8a, c) in both clones of ATP10B KO. Additionally, to distinguish between impaired differentiation and neuronal degeneration in our iPSC-derived cultures, we utilized the integrated TH-TdTomato reporter, which allows us to track the appearance and abundance of TH-positive neurons over time via FACS analysis. Our time course analysis revealed that at day 15 of differentiation, there was no significant difference in the proportion of TH-positive cells between control and KO condition, suggesting that early dopaminergic differentiation is not impaired. However, from this point onwards, a consistent reduction in TH-positive cells was observed in the KO (WT vs. clone#1 day 25 *p* = 0.0461, day 35 *p*- < 0.0001) (Fig. 8d). While these findings do not rule out subtle defects in terminal differentiation, the progressive loss of TH-positive cells following an initially normal differentiation trajectory is consistent with a degenerative process. To further support the presence of neuronal degeneration, we performed live-cell staining at day 25 using LIVE/DEAD™ Image-iT™ Caspase-3 and -7 Detection Kit. This analysis revealed a significant increase in effector caspases-3/7 activity in the KO cultures compared to controls, indicating elevated levels of apoptosis (WT vs. clone#1 *p* = 0.0130, WT vs. clone#2 *p* = 0.0252) (Fig. 8e–f).

## Discussion

ATP10B is a lysosomal transmembrane protein responsible for transporting PC and GluCer from the inner leaflet of the lysosomal membrane to the cytoplasmic side. In the context of PD, *ATP10B* has recently been recognized as a potential genetic risk factor, with loss-of-function mutations found in a subset of PD patients and decreased expression of *ATP10B* mRNA found in idiopathic PD [16]. To date, no in vivo model has been developed to explore the link of ATP10B to PD and SNpc dopaminergic neurons. To address this gap, we knocked down ATP10B in the nigral neurons of adult rats, aiming to investigate the impact of ATP10B loss-of-function on the nigrostriatal dopaminergic pathway.

We performed unilateral injections of the ATP10B knockdown AAV vectors (miR5 and miR7), together with a scrambled sequence (SCR) used as control. Analyses were conducted in two separate cohorts of rats: 1 month and 1 year post-injection. SCR, miR5, and miR7 constructs were continuously expressed through-out the course of the study, as indicated by RFP fluorescence observed at 2 weeks and 1 year post-injection in the SNpc. Importantly, we confirmed the presence of *Atp10b* RNA in rat dopaminergic neurons (Fig. S1a-b), indicating expression in this particular subtype of neurons.

Degeneration of the nigrostriatal pathway in individuals with PD results in progressive motor symptoms, including tremor, bradykinesia, akinesia, muscular rigidity, postural instability, and gait difficulties [3]. Using rodents as animal models for PD offers advantages, as degeneration in their nigrostriatal system aligns with motor impairments that can be easily evaluated through diverse behavioral tests, and experimental interventions can be more readily tested on animal models. In our experimental design, we performed unilateral injections of the vectors, enabling us to assess motor asymmetry as a read-out of unilateral neurodegeneration in the targeted SNpc and dysfunctional nigrostriatal dopaminergic neurotransmission. Notably, we found significant motor deficits in the ATP10B KD groups across a wide range of behavioral tests indicative of appearance of parkinsonian motor deficits and unilateral asymmetry in rats with decreased ATP10B expression. It is important to emphasize that in our study, we used a general neuronal promoter, which does not express the KD constructs exclusively within catecholaminergic neurons. As such, we cannot rule out the possibility that other neuronal populations in the SNpc or in neighboring transduced regions may contribute to the observed motor phenotypes. However, as further discussed below, we believe that loss of nigral dopaminergic neurons may be a major contributor.

In clinical practice, PET imaging of DAT using ^18^F-FE-PE2I serves as a valuable, cost-effective tool early diagnosis of neurodegenerative parkinsonism, including PD, is reimbursed as routine tool in several European countries including Belgium, and is used as an imaging marker in clinical trials [13, 27]. DAT PET imaging studies have revealed that PD patients show a loss of about 60% [24] of the striatal dopamine transporters by the time motor symptoms emerged, although a more recent study claimed that the loss of striatal DAT activity is about 35–45% in early PD [10]. To investigate whether the loss of ATP10B in dopaminergic neurons leads to a progressive decline in striatal dopaminergic terminals, DAT PET imaging with ^18^F-FE-PE2I was employed in a sample of rats selected randomly from the larger cohort at different time points post-injection. The binding potential of the tracer to the DAT in the dopaminergic presynaptic terminals consistently showed a decrease in the ipsilateral striatum compared to the contralateral striatum in the miR5-injected rats, suggesting a progressive loss of dopaminergic terminals. Although some further DAT downregulation is observed in human neurodegenerative Parkinsonism, its quantitative binding gives a reliable in vivo measurement of DAT availability. Because of limited access to the clinical tracer, a full longitudinal PET imaging study could not be conducted in the miR7 group. Nonetheless, we could confirm a significant decrease in the ^18^F-FE-PE2I binding in a selected group of miR7 rats at the later time points post-injection that seems to surpass the intensity of the miR5 group. Pathological examination of the brain 1 year post-injection revealed a significant loss of dopaminergic terminals (TH +) in the STR of miR5 and miR7 rats, indicating that the decrease in ^18^F-FE-PE2I binding potential occurred in parallel with a loss of striatal dopaminergic terminals.

In addition, we observed that ATP10B KD in the SNpc of rats sensitized the dopaminergic neurons to cell death at long-term. The loss of both dopaminergic neurons and terminals was notably more pronounced at 1 year post-injection compared to 1 month, suggesting a progressive rather than acute impact of ATP10B loss on cellular viability, consistent with the observations from PET imaging. A reduction in HuC/D-positive cells in the SNpc further confirmed neuronal loss in this region. In order to demonstrate similar effects in a more translational model, midbrain neuronal cultures were differentiated from two different ATP10B KO human iPSC clones. In agreement with the in vivo findings, a decrease of TH positive neurons was consistently observed in both ATP10B KO clones. Furthermore, time course analysis using a TH-tdTomato reporter revealed that dopaminergic neuron loss occurred during later stages of the differentiation protocol, indicating that ATP10B deficiency leads to a degenerative process in these cells, as observed in the rat brain.

Interestingly, ATP10B KD did not result in widespread neurodegeneration across all nigral neurons transduced by our AAV vectors in vivo. The modest magnitude of the HuC/D-positive cell loss in the SNpc suggests that non-dopaminergic neuron viability may be less affected. Also, no signs of neurodegeneration were observed in either the VTA or SNpr of the miR5 and miR7 injected rats. These results suggest that, in vivo, injection of ATP10B KD vectors primarily compromised the viability of dopaminergic neurons in the SNpc, with less impact on adjacent neuronal populations. This also highlights the importance of dopaminergic neuron loss for the observed motor deficits.

Previous studies in human cell lines have linked ATP10B KD to a loss of LAMP1 + lysosomal mass, decreased lysosomal pH, reduced lysosomal degradative capacity, and impaired lysosomal membrane integrity upon rotenone exposure. In primary neurons, ATP10B KD not only decreased lysosomal degradative capacity but also increased susceptibility to cell death [16]. Immunofluorescence staining revealed changes in late endosomal/lysosomal markers in the dopaminergic neurons of rats injected with ATP10B KD vectors at 1 year post-injection. Particularly there was a reduction in the number of late endosomes/lysosomes and the enlargement of these organelles within dopaminergic neurons of rats injected with miR7. In line with these results, LAMP1 protein levels were reduced in the midbrain differentiated culture derived from ATP10B KO iPSC clone, (Fig. S8c, f), However, the pattern observed in the miR5 group of rats was different, possibly related to the different lesion dynamics of the two vectors, so further experiments are needed to explore the link between ATP10B loss and lysosomal pathways alteration in dopaminergic neurons in vivo.

It is important to mention that, at 1 year post-injection, *Atp10b* RNA levels were significantly decreased in the transduced cells of miR7 injected rats compared to SCR, while miR5 injected rats exhibited a less pronounced downregulation of *Atp10b* RNA (Fig. S1c–d). Interestingly, these findings are consistent with the pathological differences observed between the groups: miR7 injected rats showed higher dopaminergic neurodegeneration and stronger impairment of the lysosomal markers compared to those injected with miR5, which could be due to a stronger knockdown effect obtained with miR7. Notably, although the effect measured at RNA level using miR5 was lower, this does not exclude a more significant effect at protein level. In fact, while a 50% reduction in RNA levels was observed following viral vector-mediated delivery of the miRNAs in primary neurons, the corresponding protein levels were reduced by approximately 85% [16]. Unfortunately, due to the lack of a commercially available antibody at the moment of this publication, we are currently unable to measure ATP10B protein levels in rat brain tissue by western blot or immunostaining, which is a limitation for this study.

Lysosomal activity and autophagy pathways are essential in maintaining neuronal health and proper α-synuclein levels in neurons [38]. α-Synuclein aggregation and accumulation in Lewy bodies, together with a variety of membrane components such as abnormal lysosomes or mitochondria, is one of the main hallmarks of PD [28]. Whether ATP10B loss-of-function is linked to α-synuclein accumulation and/or aggregation is still unknown. Currently there are no pathological data published from patients with *ATP10B* mutations. In our model we were unable to detect a difference in total α-synuclein levels measured by western blot in tissue extracts of the SN. Using DAB immunohistochemistry, we noted an increase of scattered Ser129-phosphorylated-α-synuclein positive cells at the injected site in the miR5 group (Fig. S9). Surprisingly, in miR7-injected rats we noted a decrease in the number of Ser129-phosphorylated-α-synuclein positive cells at the injected site*,* possibly related to the increased severity of cell loss observed in this group of animals. No effect was detected in the SNpc at 1 month post-injection. In addition, we did not find Ser129-phosphorylated-α-synuclein positive inclusions or filaments in the ipsilateral striatum at 1 month or 1 year post-injection. Based on these results, α-synuclein pathology does not appear a prominent feature in our in vivo model. While the animal model data does not indicate a strong effect on α-synuclein pathology/aggregation, we cannot exclude a primary effect of ATP10B loss-of-function on α-synuclein levels in other contexts, such as the increased levels observed in our iPSC-based model (Fig. S8c, e). It is worth noting that α-synuclein pathology exhibits heterogeneity among PD patients. It is known that individuals with specific autosomal recessive mutations, such as those in *PRKN* (PARK2) and *ATP13A2* (PARK9), often do not exhibit Lewy body formation in their brain [26]. Further research is required to understand whether loss-of-function mutations in *ATP10B* are associated with Lewy body formation.

*ATP10B* has emerged as a new potential genetic risk factor in PD and previous in vitro studies have provided insight into the function of ATP10B and the repercussions of its loss on the lysosomes and at cellular level. Additionally, disease-associated *ATP10B* variants, as well as KD of the protein, result in the loss of the ability to translocate PC and GluCer [16, 40]. Interestingly, ATP10B shares the same substrate with the enzyme GCase, encoded by *GBA*, the main genetic risk factor in PD [29]. As loss-of-function variants of both *ATP10B* and *GBA* are linked to PD risk, it is tempting to speculate whether both genes may converge on lysosomal glucosylceramide accumulation in a synergistic manner, which remains important to investigate in future studies. The metabolism of ceramides and GluCer seems to play a crucial role in PD, as evidenced by elevated levels in the plasma of sporadic PD patients, which correlate with the severity of cognitive impairment [11, 18]. In addition, not only GluCer but accumulation of other lipids has been reported in dopaminergic neurons of PD patients [4, 20].

Previously, other proteins from the P-type ATPases have been identified in a broad range of diseases [35], including loss-of-functions mutations in *ATP13A2* as cause for the severe parkinsonism Kufor-Rakeb syndrome [7, 21]. Our study establishes for the first time a link between ATP10B and PD in an in vivo model, highlighting an important role of this protein in the viability of dopaminergic neurons. Our results do not point out α-synuclein as the primary driver of the neuropathology observed in the ATP10B KD rats, suggesting that alternative pathways also play a significant role in disease development in this model. We believe that our animal model will be useful to further establish the mechanisms of cell death occurring in conditions of ATP10B loss-of-function, such as changes in lipid metabolism and lysosomal dysfunction, that may manifest in patients with ATP10B mutations. Regarding the stage of disease model, we believe that this model is useful to map processes occurring during a chronic, progressive neurodegenerative process, as evidenced by the slow progression of dopaminergic deficits illustrated by our DAT PET findings, leading to motor deficits and nigrostriatal dopaminergic neurodegeneration at 1 year post injection.

In addition, in forthcoming experiments, it will be interesting to explore in detail whether lysosomal impairments, disturbed lipid homeostasis and/or accumulation of ATP10B substrates may underlie the observed dopaminergic pathology.

## Conclusion

This is the first study to demonstrate a potential role of ATP10B in dopaminergic neurons in an in vivo model. Our results demonstrate that ATP10B depletion in SNpc neurons of adult rats triggers progressive degeneration of the nigrostriatal pathway, reflected in parkinsonian motor impairments. Furthermore, human iPSC-derived dopaminergic neurons show vulnerability to ATP10B loss, underscoring its role in maintaining dopamine neuron viability in both species. Future investigation into the molecular mechanisms disrupted upon ATP10B loss, will provide deeper insight into the pathology of the disease.

## Supplementary Information

Below is the link to the electronic supplementary material.

Supplementary file1 (TIF 40119 KB) Supplementary Fig. 1 RNAscope in situ hybridization assay for Atp10b RNA detection in 1 year post-injected rats. (a) Representative image of the SNpc of rats injected with the SCR vector 1 year post-injection, fluorescently stained with TH (green) and processed with RNAscope in situ hybridization to detect Atp10b RNA (left), or negative control probe (right). (b) Atp10b puncta quantification in TH+ cells. SCR Atp10b (n= 23), SCR Neg probe (n = 18). Each data point represents one cell. Data are mean ± SD and analyzed using Mann-Whitney t-test (**** p < 0.0001) (c) Representative image of the SNpc of rats injected with the SCR, miR5 or miR7 vector 1 year post-injection, fluorescently stained with TH (green) and RFP (yellow) antibodies, and processed with RNAscope in situ hybridization to detect Atp10b RNA (red). Cells outlined in red indicate those included in the analysis for that section. (d-e) Atp10b puncta average number per RFP+ cell. Data are mean ± s.e.m and analyzed using one-way ANOVA ( ** p < 0.01 treatment factor) and Dunnett’s post-hoc test versus SCR ( ## p < 0.01). Each data point represents the average of two sections from an individual animal. SCR (n=4), miR5 (n=4), miR7 (n=4) and negative probe control (n=1)

Supplementary file2 (TIF 12835 KB) Supplementary Fig. 2 ATP10B KD in SNpc neurons leads to motor asymmetry and decreased motor function. (a, c, g) Indicative of decreased ipsilateral dopaminergic neurotransmission, rats with ATP10B KD showed decreased preference in the use of the contralateral paw in the cylinder test (a), increased bias to ipsilateral swing in the elevated body swing test (c), and spontaneous ipsilateral turning in the open field test (g). (b) Motor coordination and balance was assessed using an accelerated rotarod test (4-40 rpm), with the average performance post-lesion normalized to the individual rat performance prior to surgery. (d, e, f) Spontaneous motor behavior was recorded during a 5-min open field test and analyzed for total distance traveled (d), velocity (e) and rearing (f). Data are mean + s.e.m. *** p < 0.001 (two-way ANOVA, treatment factor), # p < 0.05, ## p < 0.01, ### p < 0.001 (Dunnett’s post hoc test miR5 vs. SCR at corresponding time point), $ p < 0.05, $$ p < 0.01, $$$ p < 0.001 (Dunnett’s post hoc test miR7 vs. SCR at corresponding time point)

Supplementary file3 (TIF 10071 KB) Supplementary Fig. 3 Raw BPnd values from 18F-FE-PE2I PET imaging. Striatal DAT binding potential (BPnd), measured by 18F-FE-PE2I microPET imaging, in the ipsilateral (right) and the contralateral (left) striatum observed in miR5-injected animals (n=5), SCR-injected animals (n=3) and miR7-injected animals at 7-9 months (n=2) and 12 months (n=3)

Supplementary file4 (TIF 6856 KB) Supplementary Fig. 4 Pilot study: ATP10B KD leads to decreased dopaminergic terminals in the dSTR of miR5 group. TH positive area was quantified using ImageJ in 6 different sections that cover the dSTR. (a) Percentage of TH positive area ipsilateral (R) versus contralateral (L) dorsal STR of 1 year post-injection rats, SCR (n=5) and miR5 (n=7). Data are mean ± s.e.m and analyzed using t test, ** p = 0.0025. Each dot represents the average of the 6 sections per animal. (b) Representative image of TH immunohistochemical staining on 6 different sections from one SCR and miR5 rat 1 year post-injection

Supplementary file5 (TIF 14487 KB) Supplementary Fig. 5 Analysis of dopaminergic terminals in the striatum and neurons in the SNpc of 1 month and 1 year injected rats. (a-b, d-e) TH OD in the injected and non-injected dSTR and vSTR of 1 month (a-b) and 1 year (d-e) post-injection rats. (c, f) Number of TH+ cells in the injected and non-injected SNpc of 1 month (c) and 1 year (f) post-injection rats. Data are mean ± s.e.m, and analyzed using a non-parametric paired Wilcoxon signed rank test (a-c) or paired t-test (** p < 0.01, *** p < 0.001, **** p < 0.0001) (d-f). Each dot represents the average of the 7 sections per animal

Supplementary file6 (TIF 15394 KB) Supplementary Fig. 6 No significant changes in lysosomal proteins or total α-synuclein levels in whole SN protein extracts. (a-e) Protein signal values were normalized to the endogenous protein (β-actin) levels, and the ipsilateral (Ipsi) versus contralateral (Contra) percentage of the protein signal is represented. GBA signal value was obtained from the quantification of both mature and immature forms combined. (a, e) Data is analyzed using non-parametric one-way ANOVA (Kruskal Wallis) and Dunn’s post-hoc test versus SCR. (b, c, d) Data is analyzed using one-way ANOVA and Dunnett’s post-hoc test versus SCR. Data are mean ± s.e.m with each dot representing an individual animal. n=5 SCR, n=7 miR5, n=8 miR7. (f) Representative western blot images of α-synuclein and lysosomal proteins analyzed in whole SN extracts from SCR, miR5, and miR7 rats

Supplementary file7 (PNG 5987 KB) Supplementary Fig. 7 Immunofluorescent staining of late endosomal/lysosomal markers in dopaminergic neurons at 1 year post-injection. (a) Representative immunofluorescent images of LAMP1, LAMP2a, cathepsin B and GCase co-stained with TH marker in the injected and non-injected SNpc of SCR, miR5 and miR7 rats at 1 year post-injection (b-e) Density and average volume of LAMP1+, LAMP2+, CatB+, and GCase+ organelles in the injected and non-injected SNpc TH+ neurons across the different experimental groups. Data are mean ± s.e.m, and analyzed using a paired t-test comparing injected to non-injected sides within each group (* p < 0.05, ** p < 0.01, *** p < 0.001). Each dot represents the average of 3 sections per animal

Supplementary file8 (TIF 10163 KB) Supplementary Fig. 8 ATP10B KO cell lines generated in a TH-TdTomato reporter human iPSC line (BJ-SiPS). (a) Electrophoresis gel image after PCR showing the loss of a 120 bp fragment after CRISPR KO targeting exon 1 of ATP10B. (b) Sanger sequencing results confirming the deletion of the same 120 bp fragment. (c) Representative western blot images of TH, α-synuclein, LAMP1 and GAPDH proteins analyzed in WT and ATP10B KO clone #1. (d-f) Protein signal values were normalized to the endogenous protein (GAPDH) from Western Blot midbrain neuronal cultures at day 35. (d-f) Data is analyzed using non parametric unpaired t test Mann Whitney (* p < 0.05) (d-e) or parametric unpaired t test (* p < 0.05) (f). Each dot represents one independent culture

Supplementary file9 (PNG 17091 KB) Supplementary Fig. 9 Ser129-phosphorylated-α-synuclein in the SN and striatum of ATP10B KD rats. (a, b, d) The number of Ser129-phosphorylated-α-synuclein positive cells was counted throughout the injected and non-injected SN, and corresponds to an average of 3 sections per animal. Results from graph (a) belongs to the main experiment, while results in graph (b) correspond to data presented in Supplementary Fig. S4 (Pilot study). Data are mean ± s.e.m, and analyzed using a paired t-test. (c) The decrease in the number of positive cells in miR7 animals (a) may be related to the increased severity of cell loss observed in this group of animals (~65-70%; Fig. 5c), that may contribute to a general loss of positive cells. In line with this hypothesis, we noted a direct relation between the number of Ser129-phosphorylated-α-synuclein positive cells measured and the residual number of nigral TH+ cells (c; left) or striatal TH+ fibers (c; right) in miR5-injected animals at 1 year post-injection. Correlation analyses performed using Pearson correlation. (e) At 1 month post-injection, we did not observe an increase in Ser129-phosphorylated-α-synuclein positive cells in either of the experimental groups. (f-g) In addition, we did not detect the presence of Ser129-phosphorylated-α-synuclein positive inclusions or filaments in the ipsilateral striatum at 1 month (f) or 1 year (g) post-injection in either of the two KD groups. (d-e, g-f) Representative images of Ser129-phosphorylated-α-synuclein immunostaining in the SN are depicted at 1 year (d) and 1 month (e) post-injection (scale bar 500 µm, inset scale bar 50 µm), and in the striatum at 1 year (g) and 1 month (f) post-injection (scale bar 1 mm, inset scale bar 250 µm)

Supplementary file10 (DOCX 27 KB)

## Data Availability

The data, protocols, and key lab materials used and generated in this study are listed in a Key Resource Table alongside their persistent identifiers at 10.5281/zenodo.12699263. PET-CT data are available in OpenNeuro (10.18112/openneuro.ds005895.v1.0.0). Microscopy images are available in the Brain Image Library (10.35077/g.1181).

## Abbreviations

PD: Parkinson’s disease
SNpc: Substantia nigra pars compacta
SNpr: Substantia nigra pars reticulata
dSTR: Dorsal striatum
DLB: Dementia with Lewy bodies
GC: Genome copies
GluCer: Glucosylceramide
PC: Phosphatidylcholine
KD: Knockdown
GCase: Glucocerebrosidase
AAV: Adeno-associated vector
DAT: Dopamine transporter
PET: Positron emission tomography
EBST: Elevated body swing test
CT: Computed tomography
MLEM: Maximum-likelihood expectation–maximization
Hus: Hounsfield units
SRTM: Standard reference tissue model
BPnd: Binding potential
PFA: Paraformaldehyde
PBS-T: PBS with 0.1% Tergitol
RT: Room temperature
TH: Tyrosine hydroxylase
OD: Optical density
iPSCs: Induced pluripotent stem cells
KO: Knock-out
vSTR: Ventral striatum
VTA: Ventral tegmental area
