# Supplementary material for: Loss of the lysosomal lipid flippase ATP10B leads to progressive dopaminergic neurodegeneration and parkinsonian motor deficits
Source: Acta Neuropathol. 2025 Jul 17;150(1):5. doi: 10.1007/s00401-025-02908-0 (PMC12271281; doi:10.1007/s00401-025-02908-0)
Supplement: Supplementary file 1 — Supplementary file1 (TIF 40119 KB) Supplementary Fig. 1 RNAscope in situ hybridization assay for Atp10b RNA detection in 1 year post-injected rats. (a) Representative image of the SNpc of rats injected with the SCR vector 1 year post-injection, fluorescently stained with TH (green) and processed with RNAscope in situ hybridization to detect Atp10b RNA (left), or negative control probe (right). (b) Atp10b puncta quantification in TH+ cells. SCR Atp10b (n= 23), SCR Neg probe (n = 18). Each data point represents one cell. Data are mean ± SD and analyzed using Mann-Whitney t-test (**** p < 0.0001) (c) Representative image of the SNpc of rats injected with the SCR, miR5 or miR7 vector 1 year post-injection, fluorescently stained with TH (green) and RFP (yellow) antibodies, and processed with RNAscope in situ hybridization to detect Atp10b RNA (red). Cells outlined in red indicate those included in the analysis for that section. (d-e) Atp10b puncta average number per RFP+ cell. Data are mean ± s.e.m and analyzed using one-way ANOVA ( ** p < 0.01 treatment factor) and Dunnett’s post-hoc test versus SCR ( ## p < 0.01). Each data point represents the average of two sections from an individual animal. SCR (n=4), miR5 (n=4), miR7 (n=4) and negative probe control (n=1) [file 401_2025_2908_MOESM1_ESM.docx]

**Supplementary Table 1. Key Resources.**

| **RESOURCE TYPE** | **RESOURCE NAME** | **SOURCE** | **IDENTIFIER** | **ADDITIONAL INFORMATION** |
| --- | --- | --- | --- | --- |
| Dataset | Raw data for all the Figures | Zenodo | 10.5281/zenodo.12699264 |  |
| Protocol | Stereotaxic injections | protocols.io | dx.doi.org/10.17504/protocols.io.bp2l6b1qzgqe/v1 |  |
| Protocol | Rotarod test | protocols.io | dx.doi.org/10.17504/protocols.io.5qpvo3zo9v4o/v1 |  |
| Protocol | Open field test | protocols.io | dx.doi.org/10.17504/protocols.io.kxygx3dozg8j/v1 |  |
| Protocol | Cylinder test | protocols.io | dx.doi.org/10.17504/protocols.io.kxygx36zdg8j/v1 |  |
| Protocol | Elevated body swing test | protocols.io | dx.doi.org/10.17504/protocols.io.bp2l6x58zlqe/v1 |  |
| Protocol | Catalepsy test | protocols.io | dx.doi.org/10.17504/protocols.io.q26g7p24kgwz/v1 |  |
| Protocol | Small animal DAT microPET imaging | protocols.io | [dx.doi.org/10.17504/protocols.io.e6nvwdw2zlmk/v1](https://dx.doi.org/10.17504/protocols.io.e6nvwdw2zlmk/v1) |  |
| Protocol | Immunohistochemical staining, vibratome sections | protocols.io | dx.doi.org/10.17504/protocols.io.eq2lypnkqlx9/v1 |  |
| Protocol | Immunofluorescent staining ASE, vibratome sections | protocols.io | dx.doi.org/10.17504/protocols.io.n2bvj37zplk5/v1 |  |
| Protocol | Protein extraction and Western blot | protocols.io | [dx.doi.org/10.17504/protocols.io.yxmvm38q9l3p/v1](https://dx.doi.org/10.17504/protocols.io.yxmvm38q9l3p/v1) |  |
| Protocol | Immunofluorescent staining, vibratome sections | protocols.io | dx.doi.org/10.17504/protocols.io.3byl4qoxrvo5/v1 |  |
| Protocol | Generation of isogenic knockout lines | protocols.io | dx.doi.org/10.17504/protocols.io.bu7znzp6 |  |
| Protocol | iPSC midbrain differentiation in 96-well plate | protocols.io | dx.doi.org/10.17504/protocols.io.bp2l62dj5gqe/v1 |  |
| Protocol | Fixation and Immunostaining protocol midbrain culture | protocols.io | [dx.doi.org/10.17504/protocols.io.j8nlk8wowl5r/v1](https://dx.doi.org/10.17504/protocols.io.j8nlk8wowl5r/v1) |  |
| Protocol | RNAScope in tissue sections | protocols.io | [dx.doi.org/10.17504/protocols.io.14egn3odml5d/v1](https://dx.doi.org/10.17504/protocols.io.14egn3odml5d/v1) |  |
| Antibody | Rabbit anti-TH | Millipore | Cat# AB152, RRID: AB_390204 | 1:10.000 IHC |
| Antibody | Chicken anti-TH | Aves Lab | Cat# TYH, RRID: AB_10013440 | 1:1000 IHC |
| Antibody | Rabbit anti-RFP | Rockland | Cat# 600-401-379S, RRID: AB_11182807 | 1:500 IHC |
| Antibody | Rat anti-LAMP1 | Novus | Cat# NBP1-49151, RRID: AB_10011343 | 1:500 IHC |
| Antibody | Rabbit anti-GBA C-terminal | Sigma-Aldrich | Cat# G4171, RRID: AB_1078958 | 1:500 IHC, 1:1000 WB |
| Antibody | Goat anti-Cathepsin B | R&D Systems | Cat# AF965, RRID: AB_2086949 | 1:500 IHC, 1:1000 WB |
| Antibody | Rat anti-LAMP2a | Abcam | Cat# ab13524, RRID: AB_2134736 | 1:500 IHC |
| Antibody | Mouse anti-HuC/D | Invitrogen | Cat# A-21271, RRID: AB_221448), | 1:500 IHC |
| Antibody | Rabbit anti-LAMP1 | Abcam | Cat# ab25630, RRID: AB_470708 | 1:1000 WB |
| Antibody | Rabbit anti-P62/SQTM1 | Proteintech | Cat# 55274-1-AP, RRID: AB_11182278 | 1:1000 WB |
| Antibody | Mouse anti-α-synuclein | BD Bioscience | Cat# 610786, RRID: AB_398107 | 1:1000 WB |
| Antibody | Mouse anti-Bactin | Sigma Aldrich | Cat# A5441, RRID: AB_476744 | 1:1000 WB |
| Antibody | Mouse anti-TH | Millipore | Cat#MAB318; RRID: AB_2201528 | 1:300 ICC |
| Antibody | Chicken anti-MAP2 | BioLegend | Cat# 822501, RRID: AB_2564858) | 1:400 ICC |
| Antibody | Biotinylated goat anti-rabbit IgG | DakoCytomation | Cat# ab6720, RRID: AB_954902 | 1:1000 IHC |
| Antibody | Donkey anti-chicken Alexa 488 | Jackson ImmunoResearch Labs | Cat# 703-545-155, RRID: AB_2340375 | 1:500 IHC |
| Antibody | Donkey anti-chicken Alexa 555 | Thermo Fisher Scientific | Cat# A78949, RRID: AB_2921071 | 1:500 IHC |
| Antibody | Donkey anti-rabbit Alexa 555 | Thermo Fisher Scientific | Cat# A-31572, RRID: AB_162543 | 1:500 IHC |
| Antibody | Donkey anti-rabbit Alexa 647 | Thermo Fisher Scientific | Cat# A32795, RRID: AB_2762835 | 1:500 IHC |
| Antibody | Donkey anti-goat Alexa 647 | Thermo Fisher Scientific | Cat# A-21447, RRID: AB_2535864 | 1:500 IHC |
| Antibody | Donkey anti-rat Alexa 488 | Abcam | Cat# ab150153, RRID: AB_2737355 | 1:500 IHC |
| Antibody | Donkey anti-mouse Alexa 647 | Invitrogen | Cat# A31571, RRID: AB_162542) |  |
| Antibody | Goat anti-rabbit horseradish peroxidase-conjugated secondary antibody | Agilent | Cat# P0448, RRID: AB_2617138 | 1:10.000 WB |
| Antibody | Goat anti-mouse horseradish peroxidase-conjugated secondary antibody | Agilent | Cat# P0447, RRID: AB_2617137 | 1:10.000 WB |
| Antibody/chemical | Streptavidin/HRP | Agilent | Cat# P039701-2 | 1:1000 IHC |
| Antibody | Donkey anti-Mouse IgG, Alexa Fluor 555 | Invitrogen | Cat# A-31570, RRID :AB_2536180 | 1:500 ICC |
| Antibody | Goat anti-Chicken IgY (H+L) Alexa Fluor 488 | Thermo Fisher Scientific | Cat# A-11039, RRID: AB_2534096 | 1:500 ICC |
| Recombinant DNA | CMVenhanced-Synapsin-mCherry-SCR | Addgene plasmid | RRID: Addgene_216393 |  |
| Recombinant DNA | CMVenhanced-Synapsin-mCherry-miR5 | Addgene plasmid | RRID: Addgene_216391 |  |
| Recombinant DNA | CMVenhanced-Synapsin-mCherry-miR7 | Addgene plasmid | RRID: Addgene_216392 |  |
| Chemical, peptide, or recombinant protein | RNAscope™ Probe - Rn-Atp10b-C1 | Advanced Cell Diagnostics | REF: 1155561-C1 |  |
| Chemical, peptide, or recombinant protein | RNAscope™ 3-plex Negative Control Probe | Advanced Cell Diagnostics | REF: 320871 |  |
| Chemical, peptide, or recombinant protein | RNAscope™ Multiplex Fluorescent Reagent Kit v2 with TSA Vivid Dyes | Advanced Cell Diagnostics | REF: 323270 |  |
| Chemical, peptide, or recombinant protein | ^18^F-FE-PE2I | UZ Leuven, Leuven, Belgium |  | Obtained from the hospital radiopharmacy at UZ Leuven |
| Chemical, peptide, or recombinant protein | DAB 3,3'diaminobenzidine tetrahydrodhloride | Sigma | D5905 |  |
| Chemical, peptide, or recombinant protein | Criterion™ Tris-HCl Protein Gel | Bio-Rad | 3450027 |  |
| Chemical, peptide, or recombinant protein | PVDF membrane | Bio-Rad | 1620174 |  |
| Chemical, peptide, or recombinant protein | Clarity Western ECL | Bio-Rad | 1705061 |  |
| Chemical, peptide, or recombinant protein | ECL^™^ Select Western Blotting Detection Reagent | Cytiva | GERPN2235 |  |
| Experimental model: Cell line | ATP10B KO clone#1 |  | RRID: CVCL_E3SX |  |
| Experimental model: Cell line | ATP10B KO clone#2 |  | RRID: CVCL_E3SY |  |
| Experimental model: Cell line | BJ SiPS-D TH-TdTomato |  | RRID: CVCL_C8FN |  |
| Experimental model: Rats | Han:WI (HanRj:WI) | Janvier | RRID: RGD_13792727 |  |
| Software | Animal Tracker |  | RRID: SCR_014397 | http://animaltracker.elte.hu |
| Software | QuPath |  | RRID: SCR_018257 | https://qupath.github.io |
| Software | GraphPad Prism |  | RRID: SCR_002798 | http://www.graphpad.com |
| Software | Imaris | Oxford Instruments | RRID: SCR_007370 | http://www.bitplane.com/imaris/imaris/ |
| Software | Image Quant |  | RRID: SCR_014246 | http://www.gelifesciences.com |
| Software | Cell Profiler |  | RRID: SCR_007358 | http://cellprofiler.org |
| Software | PKIN |  | RRID: SCR_016547 | https://www.pmod.com |
