# Supplementary figures and images for: Loss of the lysosomal lipid flippase ATP10B leads to progressive dopaminergic neurodegeneration and parkinsonian motor deficits
Source: Acta Neuropathol. 2025 Jul 17;150(1):5. doi: 10.1007/s00401-025-02908-0 (PMC12271281; doi:10.1007/s00401-025-02908-0)

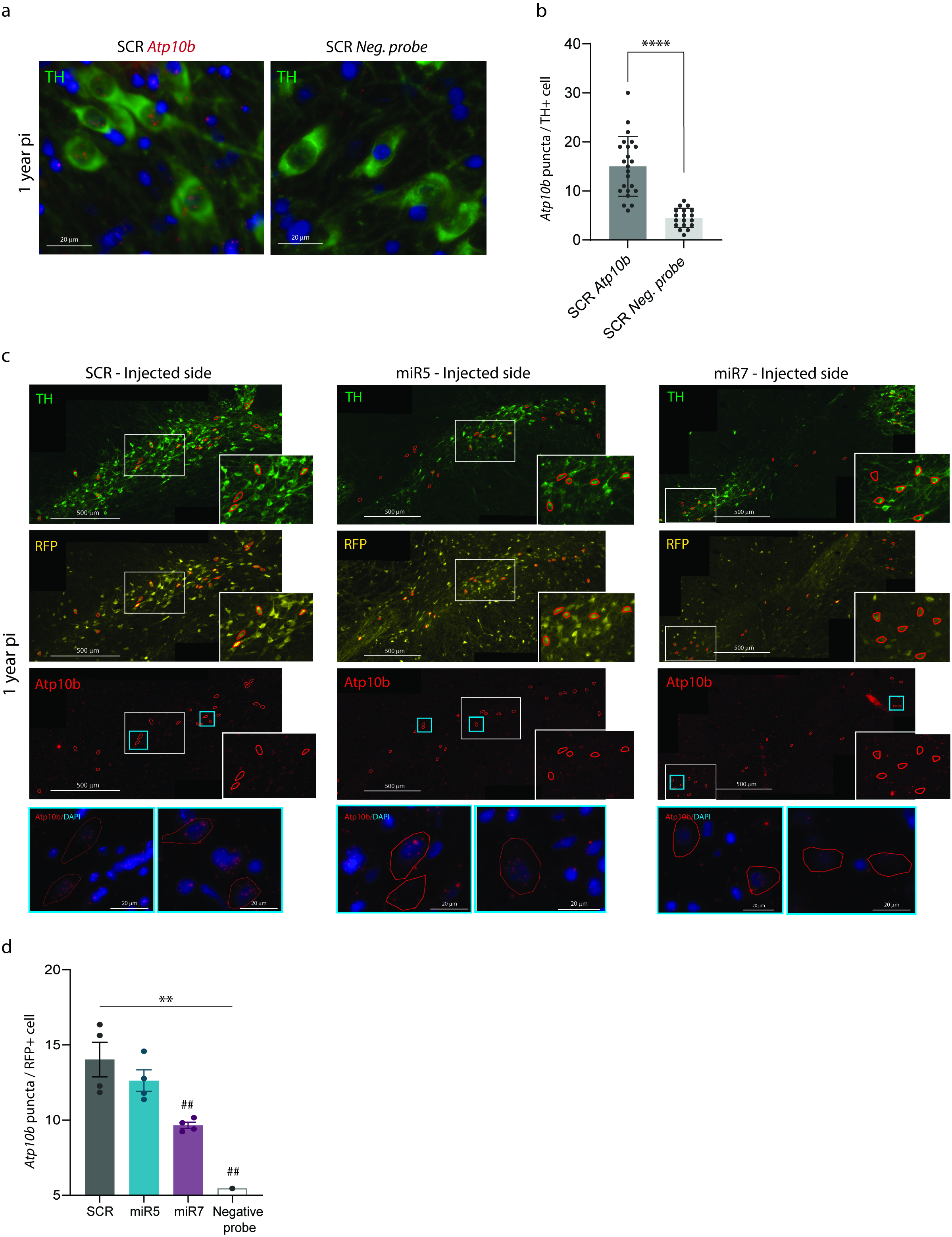

Supplement: Supplementary file 2 — Supplementary file2 (TIF 12835 KB) Supplementary Fig. 2 ATP10B KD in SNpc neurons leads to motor asymmetry and decreased motor function. (a, c, g) Indicative of decreased ipsilateral dopaminergic neurotransmission, rats with ATP10B KD showed decreased preference in the use of the contralateral paw in the cylinder test (a), increased bias to ipsilateral swing in the elevated body swing test (c), and spontaneous ipsilateral turning in the open field test (g). (b) Motor coordination and balance was assessed using an accelerated rotarod test (4-40 rpm), with the average performance post-lesion normalized to the individual rat performance prior to surgery. (d, e, f) Spontaneous motor behavior was recorded during a 5-min open field test and analyzed for total distance traveled (d), velocity (e) and rearing (f). Data are mean + s.e.m. *** p < 0.001 (two-way ANOVA, treatment factor), # p < 0.05, ## p < 0.01, ### p < 0.001 (Dunnett’s post hoc test miR5 vs. SCR at corresponding time point), $ p < 0.05, $$ p < 0.01, $$$ p < 0.001 (Dunnett’s post hoc test miR7 vs. SCR at corresponding time point) [file 401_2025_2908_MOESM2_ESM.tif]

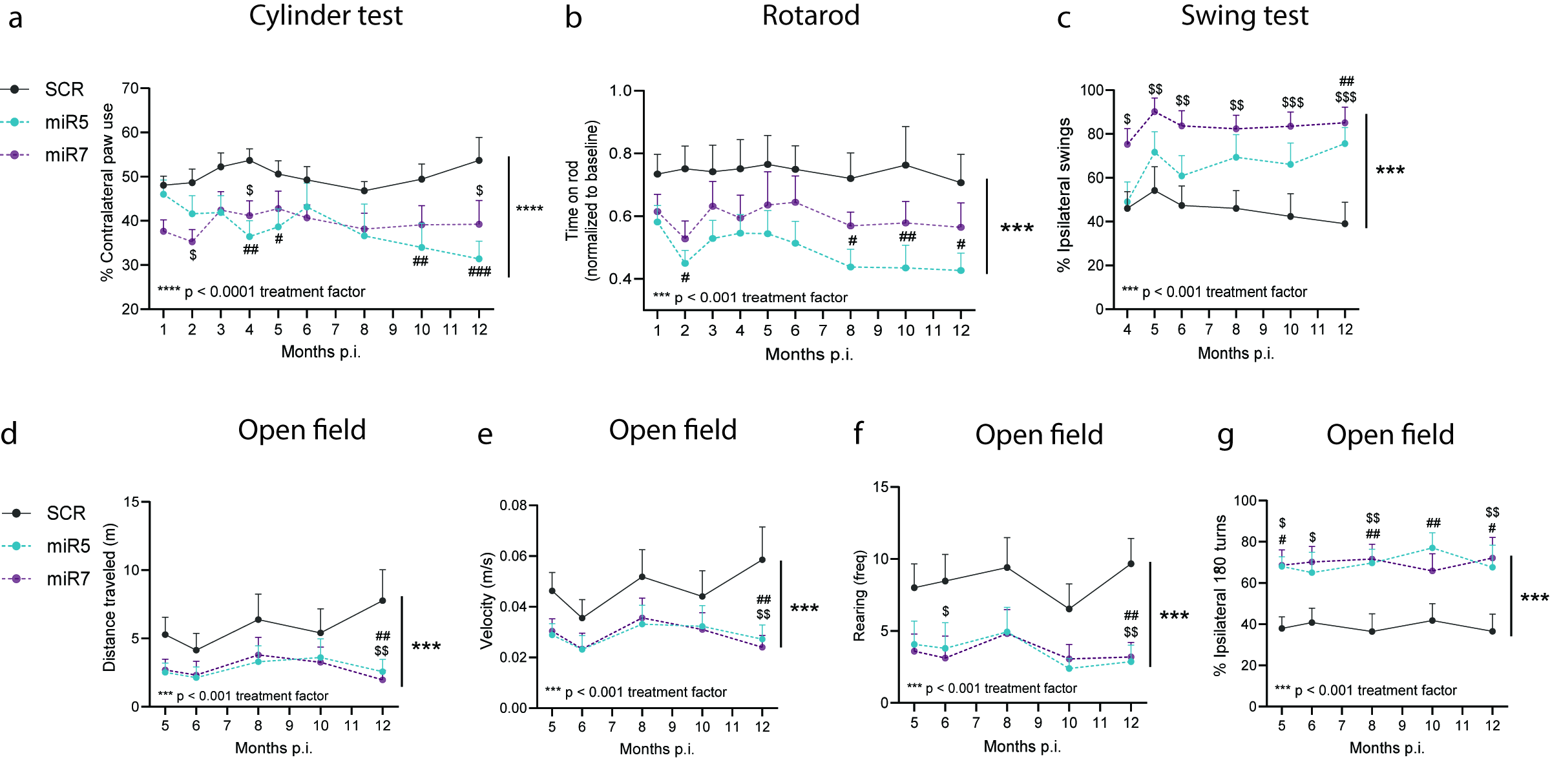

Supplement: Supplementary file 3 — Supplementary file3 (TIF 10071 KB) Supplementary Fig. 3 Raw BPnd values from 18F-FE-PE2I PET imaging. Striatal DAT binding potential (BPnd), measured by 18F-FE-PE2I microPET imaging, in the ipsilateral (right) and the contralateral (left) striatum observed in miR5-injected animals (n=5), SCR-injected animals (n=3) and miR7-injected animals at 7-9 months (n=2) and 12 months (n=3) [file 401_2025_2908_MOESM3_ESM.tif]

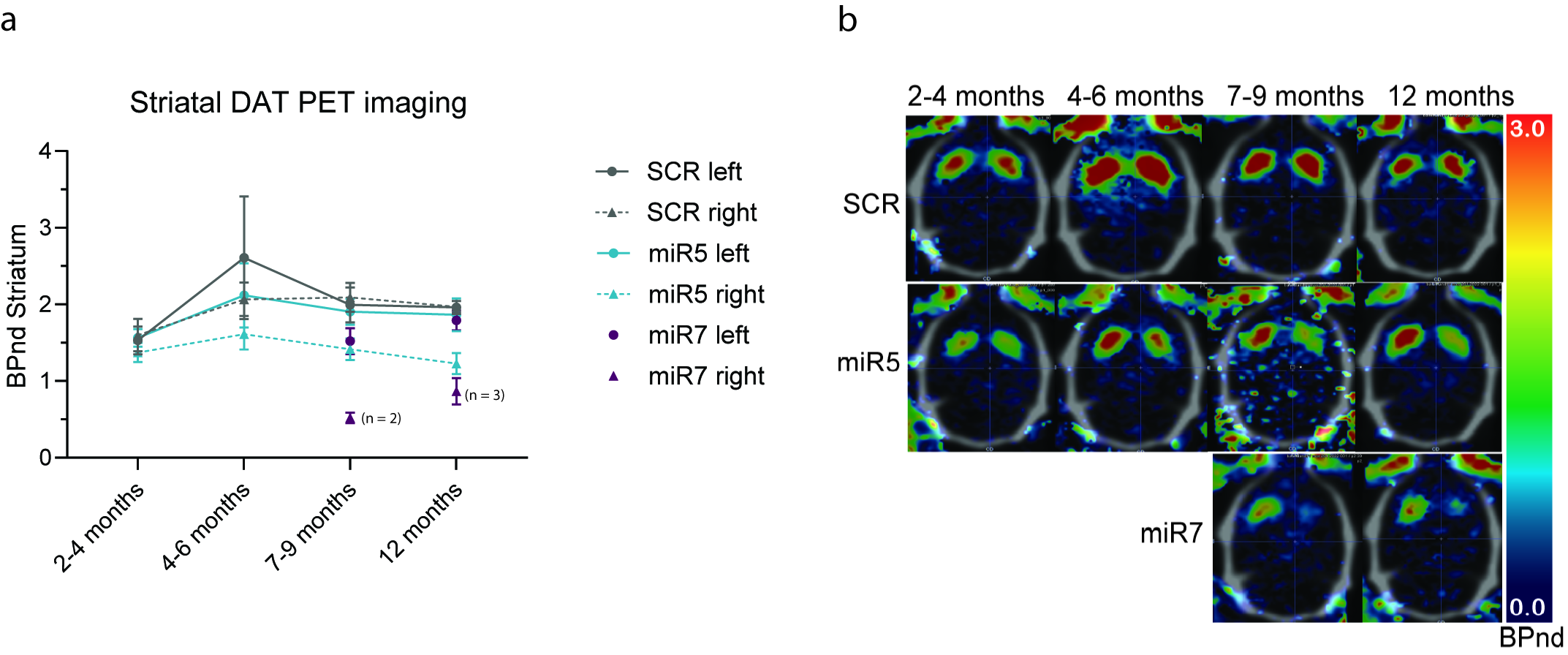

Supplement: Supplementary file 4 — Supplementary file4 (TIF 6856 KB) Supplementary Fig. 4 Pilot study: ATP10B KD leads to decreased dopaminergic terminals in the dSTR of miR5 group. TH positive area was quantified using ImageJ in 6 different sections that cover the dSTR. (a) Percentage of TH positive area ipsilateral (R) versus contralateral (L) dorsal STR of 1 year post-injection rats, SCR (n=5) and miR5 (n=7). Data are mean ± s.e.m and analyzed using t test, ** p = 0.0025. Each dot represents the average of the 6 sections per animal. (b) Representative image of TH immunohistochemical staining on 6 different sections from one SCR and miR5 rat 1 year post-injection [file 401_2025_2908_MOESM4_ESM.tif]

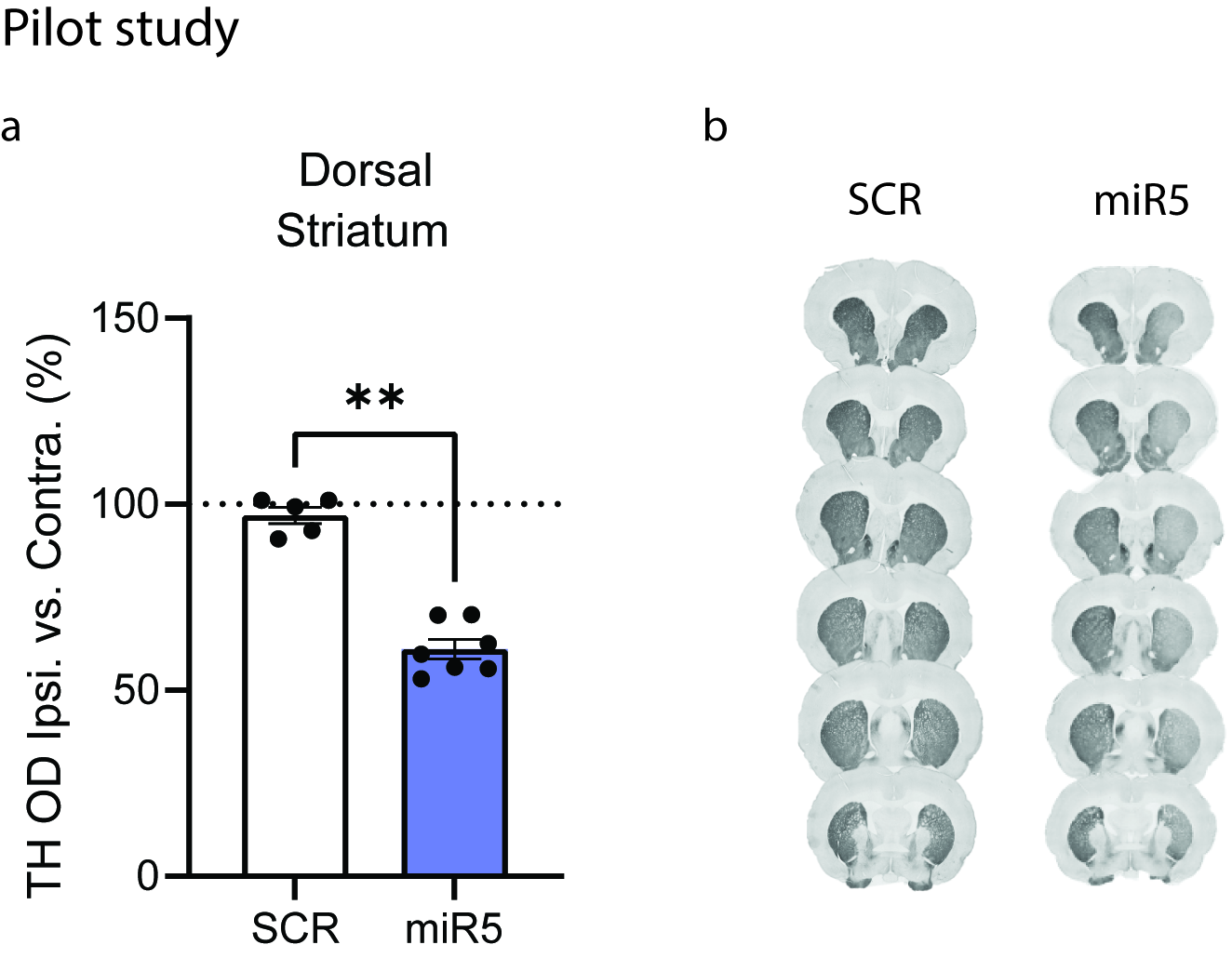

Supplement: Supplementary file 5 — Supplementary file5 (TIF 14487 KB) Supplementary Fig. 5 Analysis of dopaminergic terminals in the striatum and neurons in the SNpc of 1 month and 1 year injected rats. (a-b, d-e) TH OD in the injected and non-injected dSTR and vSTR of 1 month (a-b) and 1 year (d-e) post-injection rats. (c, f) Number of TH+ cells in the injected and non-injected SNpc of 1 month (c) and 1 year (f) post-injection rats. Data are mean ± s.e.m, and analyzed using a non-parametric paired Wilcoxon signed rank test (a-c) or paired t-test (** p < 0.01, *** p < 0.001, **** p < 0.0001) (d-f). Each dot represents the average of the 7 sections per animal [file 401_2025_2908_MOESM5_ESM.tif]

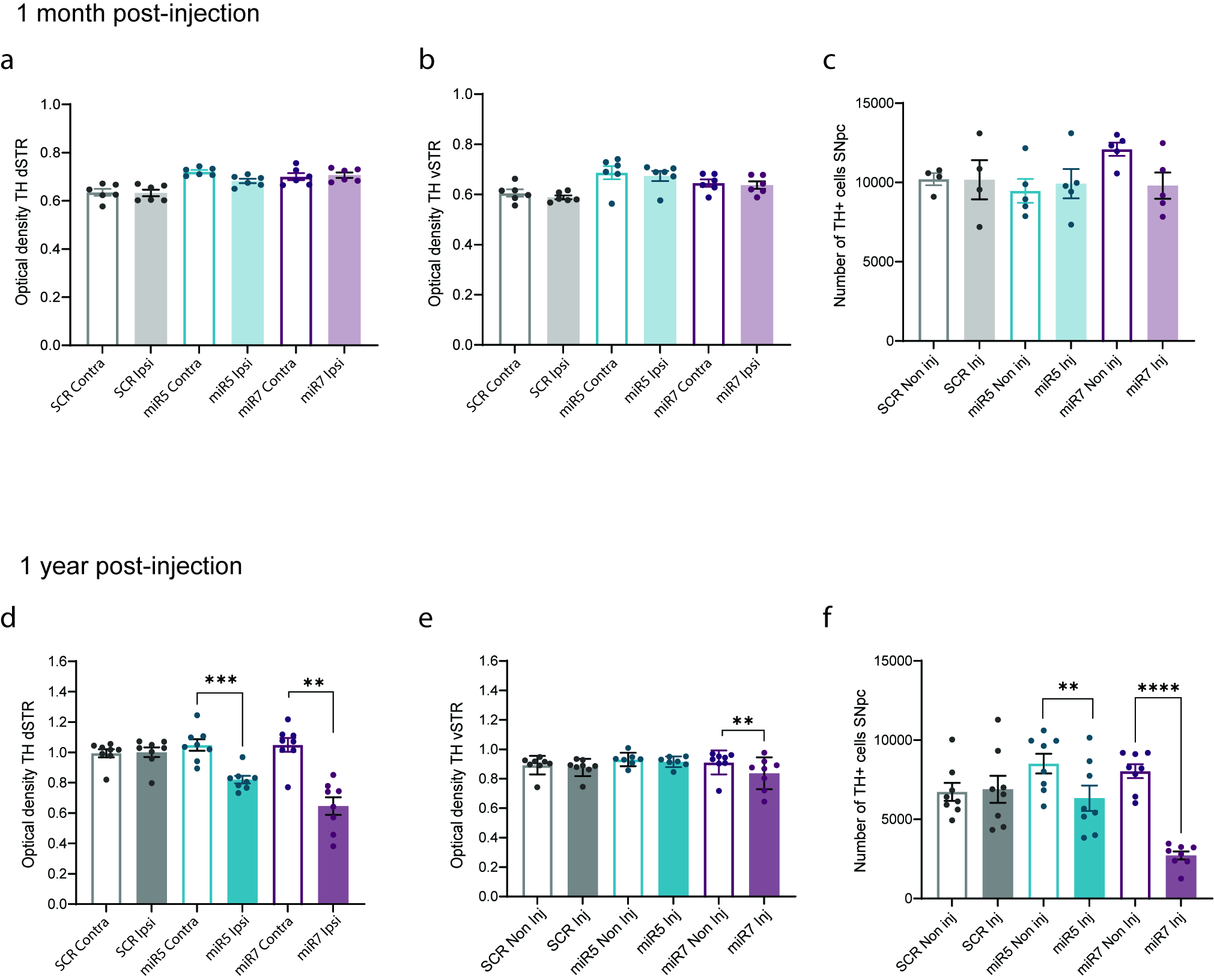

Supplement: Supplementary file 6 — Supplementary file6 (TIF 15394 KB) Supplementary Fig. 6 No significant changes in lysosomal proteins or total α-synuclein levels in whole SN protein extracts. (a-e) Protein signal values were normalized to the endogenous protein (β-actin) levels, and the ipsilateral (Ipsi) versus contralateral (Contra) percentage of the protein signal is represented. GBA signal value was obtained from the quantification of both mature and immature forms combined. (a, e) Data is analyzed using non-parametric one-way ANOVA (Kruskal Wallis) and Dunn’s post-hoc test versus SCR. (b, c, d) Data is analyzed using one-way ANOVA and Dunnett’s post-hoc test versus SCR. Data are mean ± s.e.m with each dot representing an individual animal. n=5 SCR, n=7 miR5, n=8 miR7. (f) Representative western blot images of α-synuclein and lysosomal proteins analyzed in whole SN extracts from SCR, miR5, and miR7 rats [file 401_2025_2908_MOESM6_ESM.tif]

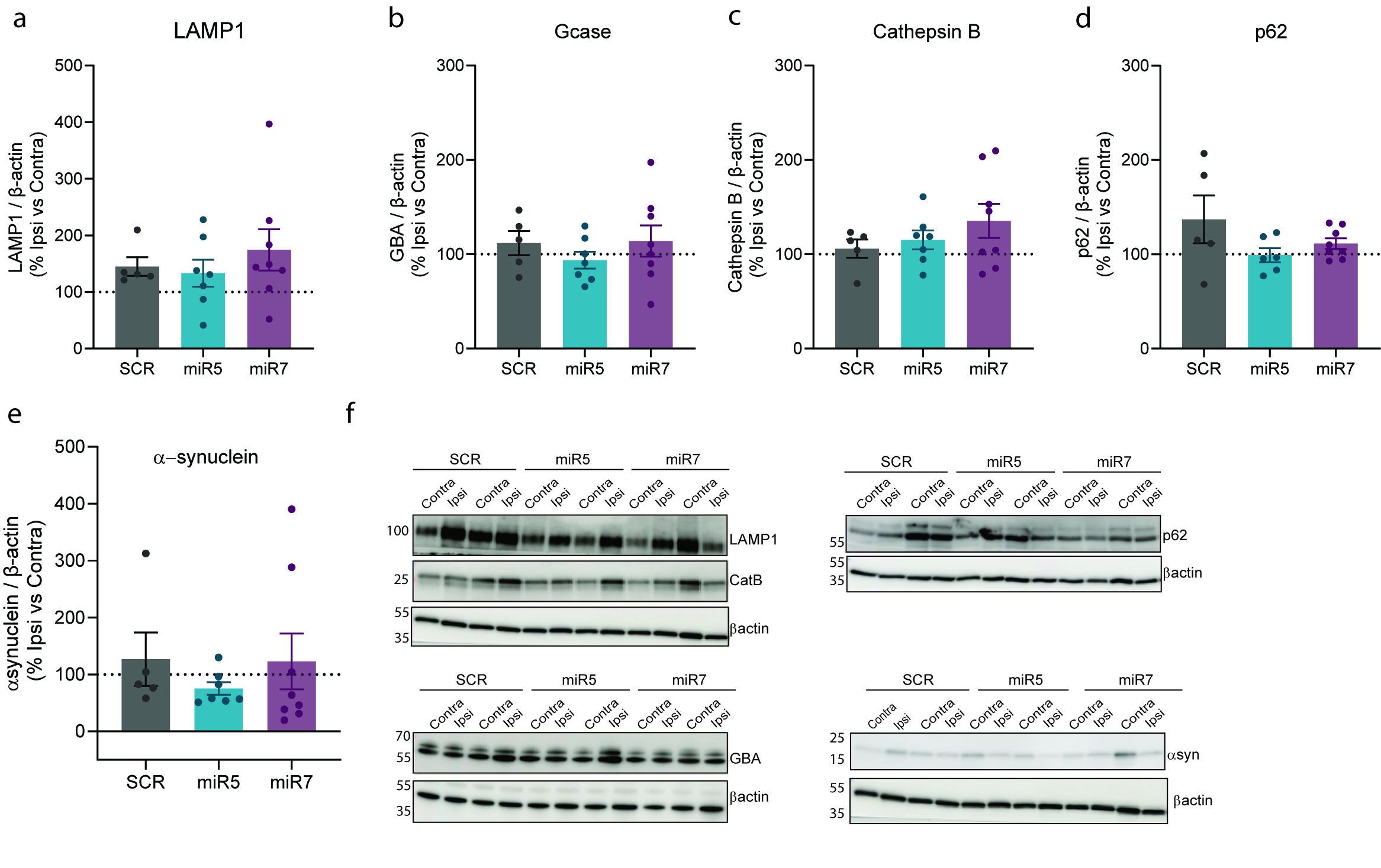

Supplement: Supplementary file 7 — Supplementary file7 (PNG 5987 KB) Supplementary Fig. 7 Immunofluorescent staining of late endosomal/lysosomal markers in dopaminergic neurons at 1 year post-injection. (a) Representative immunofluorescent images of LAMP1, LAMP2a, cathepsin B and GCase co-stained with TH marker in the injected and non-injected SNpc of SCR, miR5 and miR7 rats at 1 year post-injection (b-e) Density and average volume of LAMP1+, LAMP2+, CatB+, and GCase+ organelles in the injected and non-injected SNpc TH+ neurons across the different experimental groups. Data are mean ± s.e.m, and analyzed using a paired t-test comparing injected to non-injected sides within each group (* p < 0.05, ** p < 0.01, *** p < 0.001). Each dot represents the average of 3 sections per animal [file 401_2025_2908_MOESM7_ESM.tif]

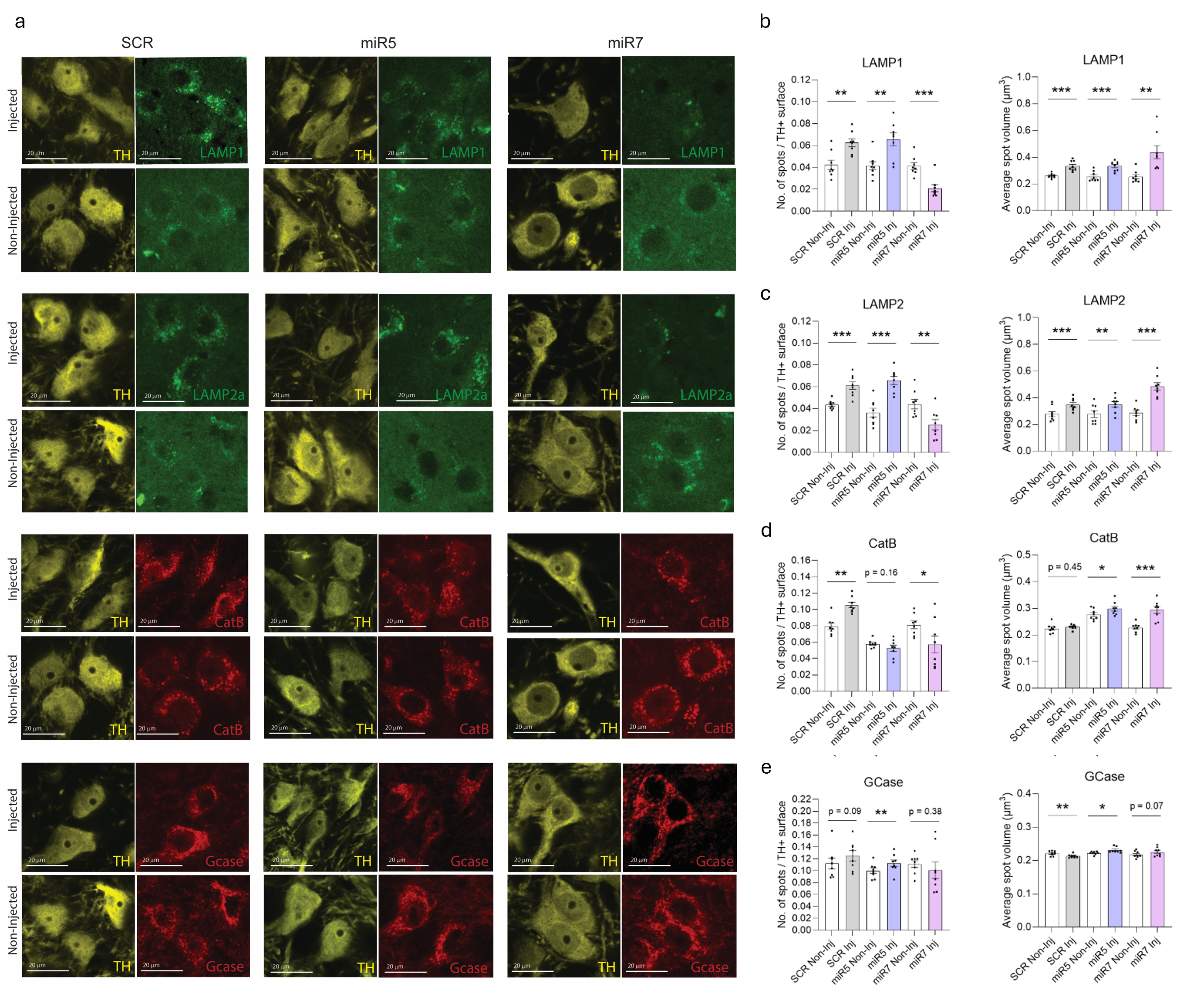

Supplement: Supplementary file 8 — Supplementary file8 (TIF 10163 KB) Supplementary Fig. 8 ATP10B KO cell lines generated in a TH-TdTomato reporter human iPSC line (BJ-SiPS). (a) Electrophoresis gel image after PCR showing the loss of a 120 bp fragment after CRISPR KO targeting exon 1 of ATP10B. (b) Sanger sequencing results confirming the deletion of the same 120 bp fragment. (c) Representative western blot images of TH, α-synuclein, LAMP1 and GAPDH proteins analyzed in WT and ATP10B KO clone #1. (d-f) Protein signal values were normalized to the endogenous protein (GAPDH) from Western Blot midbrain neuronal cultures at day 35. (d-f) Data is analyzed using non parametric unpaired t test Mann Whitney (* p < 0.05) (d-e) or parametric unpaired t test (* p < 0.05) (f). Each dot represents one independent culture [file 401_2025_2908_MOESM8_ESM.png]

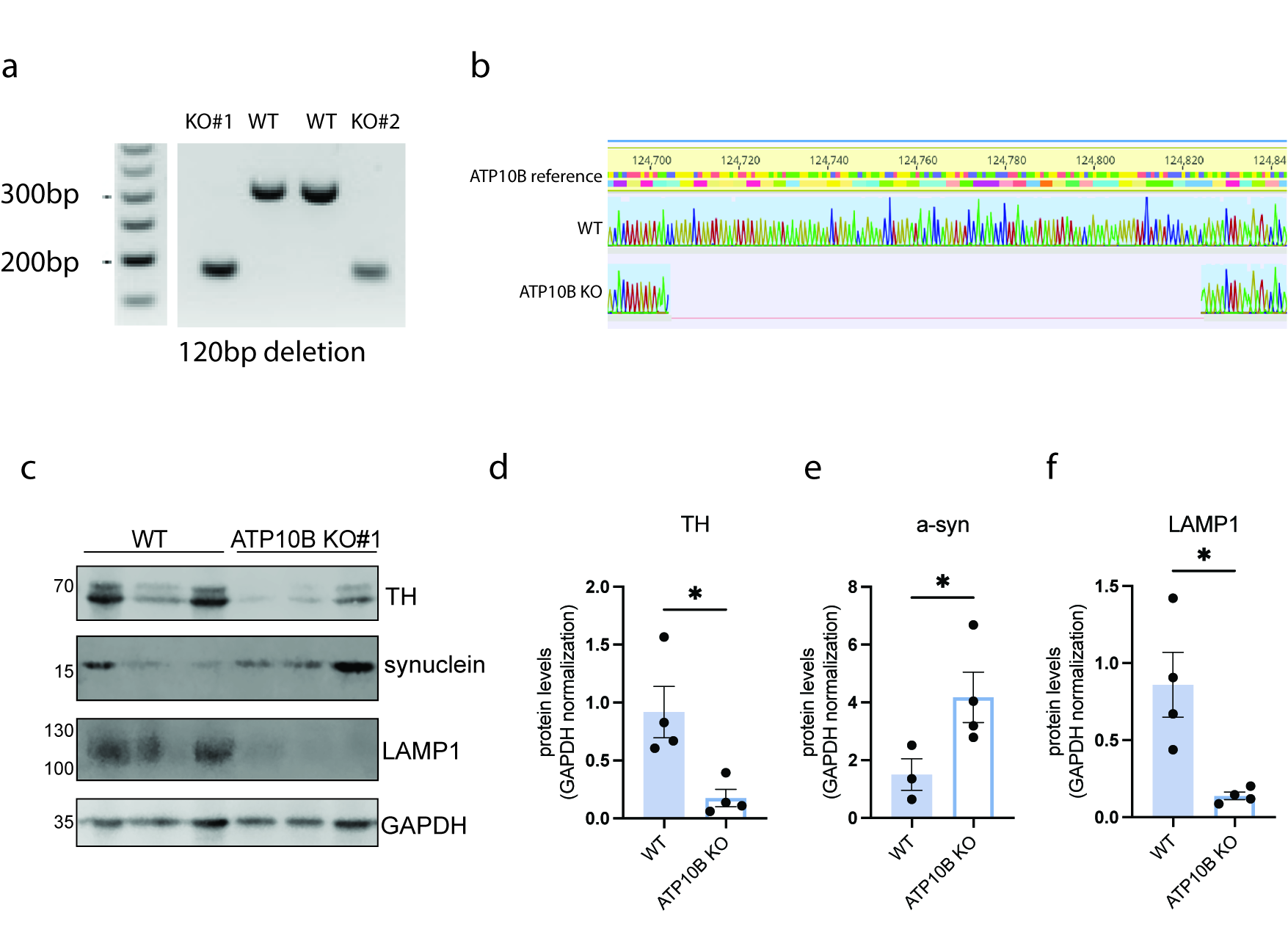

Supplement: Supplementary file 9 — Supplementary file9 (PNG 17091 KB) Supplementary Fig. 9 Ser129-phosphorylated-α-synuclein in the SN and striatum of ATP10B KD rats. (a, b, d) The number of Ser129-phosphorylated-α-synuclein positive cells was counted throughout the injected and non-injected SN, and corresponds to an average of 3 sections per animal. Results from graph (a) belongs to the main experiment, while results in graph (b) correspond to data presented in Supplementary Fig. S4 (Pilot study). Data are mean ± s.e.m, and analyzed using a paired t-test. (c) The decrease in the number of positive cells in miR7 animals (a) may be related to the increased severity of cell loss observed in this group of animals (~65-70%; Fig. 5c), that may contribute to a general loss of positive cells. In line with this hypothesis, we noted a direct relation between the number of Ser129-phosphorylated-α-synuclein positive cells measured and the residual number of nigral TH+ cells (c; left) or striatal TH+ fibers (c; right) in miR5-injected animals at 1 year post-injection. Correlation analyses performed using Pearson correlation. (e) At 1 month post-injection, we did not observe an increase in Ser129-phosphorylated-α-synuclein positive cells in either of the experimental groups. (f-g) In addition, we did not detect the presence of Ser129-phosphorylated-α-synuclein positive inclusions or filaments in the ipsilateral striatum at 1 month (f) or 1 year (g) post-injection in either of the two KD groups. (d-e, g-f) Representative images of Ser129-phosphorylated-α-synuclein immunostaining in the SN are depicted at 1 year (d) and 1 month (e) post-injection (scale bar 500 µm, inset scale bar 50 µm), and in the striatum at 1 year (g) and 1 month (f) post-injection (scale bar 1 mm, inset scale bar 250 µm) [file 401_2025_2908_MOESM9_ESM.tif]

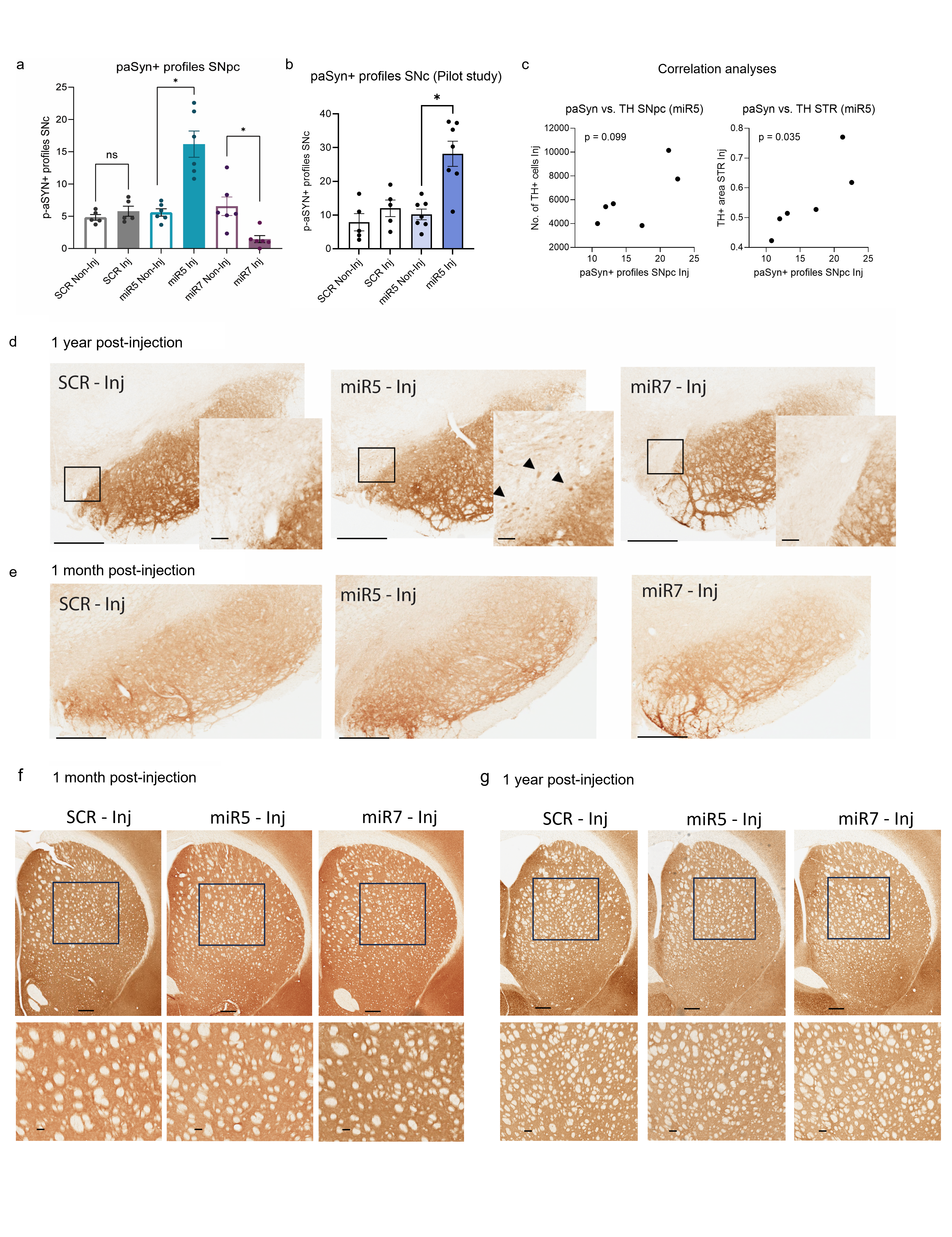

Supplement: Supplementary file 10 — Supplementary file10 (DOCX 27 KB) [file 401_2025_2908_MOESM10_ESM.png]
